# Recent Advances in Combining Waterborne Acrylic Dispersions with Biopolymers

**DOI:** 10.3390/polym17081027

**Published:** 2025-04-10

**Authors:** Jordi Solera-Sendra, Nicholas Ballard, Luis J. del Valle, Lourdes Franco

**Affiliations:** 1 eb-Policom—PSEP, Departament d’Enginyeria Química, Escola d’Enginyeria de Barcelona Est (EEBE), Universitat Politècnica de Catalunya, BarcelonaTech (UPC), Av. Eduard Maristany 16, 08019 Barcelona, Spain; jordi.solera@upc.edu (J.S.-S.); luis.javier.del.valle@upc.edu (L.J.d.V.); 2 Barcelona Research Centre in Multiscale Science and Engineering, Universitat Politècnica de Catalunya, BarcelonaTech (UPC), Av. Eduard Maristany, 16, 08019 Barcelona, Spain; 3 Applications Department, Lubrizol Advanced Materials, Camí de Can Calders, 13, 08173 Sant Cugat del Vallès, Spain; 4 Polymat, University of the Basque Country UPV/EHU, Tolosa Hiribidea 72, 20018 Donostia-San Sebastián, Spain; nicholas.ballard@polymat.eu; 5 Ikerbasque, Basque Foundation for Science, 48013 Bilbao, Spain

**Keywords:** waterborne acrylic latexes, biopolymers, casein, collagen, soy protein, cellulose, nanocellulose, starch, chitin, chitosan

## Abstract

Water-based (meth)acrylic (co)polymer dispersions are produced on a large scale for various applications including coatings, adhesives, paints, and construction materials. A major benefit of waterborne polymer dispersions as compared to more traditional solvent-based alternatives is the low volatile organic compound (VOC) content, which results in an improved environmental profile. Following the trend of sustainability that has driven the growth of acrylic dispersions, recent research has focused on further enhancing the properties of these products by incorporating biobased materials such as polysaccharides (e.g., cellulose, starch, chitin, and chitosan), and proteins (e.g., casein, soy protein, and collagen). Amongst a large number of benefits, the incorporation of biomaterials can serve to decrease the amount of petroleum-based polymers in the formulation and can also contribute to enhance the physical properties of the resulting bio-composites. In this review, the beneficial role of these biopolymers when combined with waterborne acrylic systems is summarized. Recent advances in the use of these biobased and biodegradable materials are covered, aiming to provide guidance for the development of more sustainable, high-performance latex-based bio-composites with minimal environmental impact.

## 1. Introduction

Acrylic polymers, which include both acrylates and methacrylates, play a crucial role in a wide range of industrial applications due to their excellent mechanical, optical, and chemical properties [1,2]. These polymers are extensively used in various sectors such as paints [3], coatings [4], adhesives [5,6], textiles [7], and construction [5,8], as a result of their versatility, transparency, durability and weather resistance. Acrylic polymers can be synthesized through a number of polymerization techniques, including bulk and solution-based polymerization. However, these conventional methods present certain drawbacks, such as the formation of high-viscosity mixtures and the use of organic solvents, which leads to the emission of volatile organic compounds (VOCs), posing significant health and environmental risks [9,10]. As an alternative, emulsion polymerization (EP), a technique that uses water as a reaction media, can be used. This offers a more sustainable alternative to solution based polymerization processes and has become a major component of the market for acrylic polymers [11]. The emulsion polymerization technique offers several additional advantages, such as enhanced safety, improved reaction control, relatively low viscosities, better heat dissipation, and the elimination of VOCs [12]. These benefits make emulsion polymerization the preferred method for large-scale production of acrylic polymers for many applications.

Despite the relatively favorable environmental profile of emulsion polymerization, as the chemical industry moves toward more sustainable practices, there is increasing pressure to further reduce the environmental impact of acrylic polymers. In particular, there is a growing desire to reduce our reliance on petroleum-based polymers and adopt eco-friendly alternatives. One significant advance in this direction is the incorporation of biopolymers into acrylic matrices. The resulting hybrid materials can not only result in an improvement in the physical properties of acrylic polymers but also improve biodegradability while reducing the amount of petroleum-based chemicals in the final product.

In the following review, the potential use of biomaterials to improve the characteristics of acrylic polymer dispersions is covered. After a brief description of the emulsion polymerization process, the characteristics of the most widely produced biopolymers will be discussed, followed by an analysis of their main structural characteristics and properties. The review will then explore the synthetic methods employed to incorporate biopolymers into waterborne acrylic latexes, including both ex situ approaches, such as blending the biopolymer with pre-formed acrylic latex, and in situ polymerization, where polymerization occurs in the presence of the biopolymer. Key modifications and functionalization techniques, such as grafting, will also be highlighted showing its impact on the compatibility between the biopolymer and synthetic acrylates. Finally, the properties of the resulting hybrid materials will be discussed, focusing on their potential use in industrial applications. Particular emphasis will be placed on the advantages offered by these hybrid systems, including enhanced bio-content, improved sustainability, and physical properties. This structured analysis aims to provide a clear and comprehensive perspective on the role of each biopolymer in acrylic latex systems, highlighting their potential to advance the development of sustainable polymeric materials.

## 2. Waterborne Acrylic Latexes

**Figure 1:**
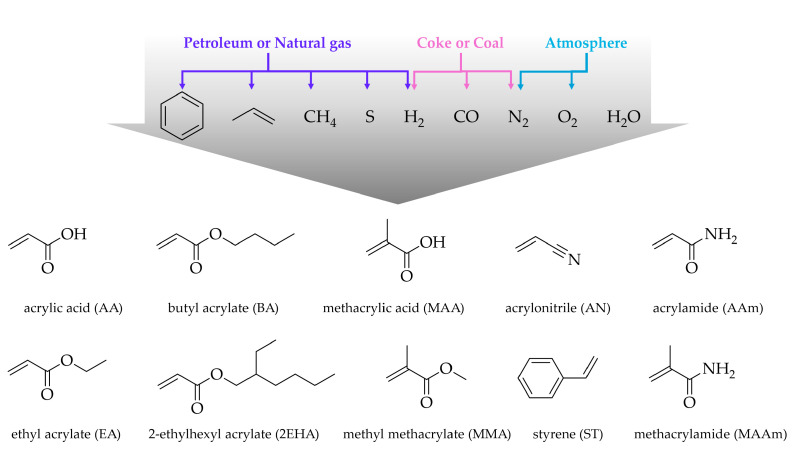
Chemical structure of acrylic acid, methacrylic acid, their ester derivatives, and some common vinyl comonomers used in waterborne acrylic latexes such as acrylonitrile, styrene, acrylamide, and methacrylamide. At the top, the principal compounds and main sources for their synthesis are indicated.

Waterborne acrylic latexes are two-phase systems, where polymeric particles are dispersed within a continuous aqueous phase. The widespread use of acrylic latexes is attributed to their cost effectiveness and the versatile properties that can be achieved by tailoring monomer composition [2]. The versatility arises from the wide range of available monomers, enabling fine-tuned control over the polymer’s physical and chemical properties (Figure 1).

Acrylates, which are esters derived from acrylic acid (AA), represent a large family of compounds due to the vast number of functional groups they can incorporate. Among them, we can highlight ethyl acrylate (EA), butyl acrylate (BA), and 2-ethylhexyl acrylate (2EHA). These are classified as “soft” monomers due to the low glass transition temperature (T_g_) of their corresponding homopolymer, which impart flexibility, elasticity, and softness to the resulting polymers, that are commonly desired for coatings, adhesives, and sealants.

Methacrylates are the ester derivatives of methacrylic acid (MAA). Methyl methacrylate (MMA) is the most widely used monomer of this family. MMA, in contrast to the aforementioned monomers, is categorized as a “hard” monomer, due to the high T_g_ of its homopolymer. Its incorporation increases the rigidity, hardness, and chemical resistance, which are important properties for applications such as coatings and structural adhesives.

AA is primarily produced through the oxidation of propylene, and it serves as a key precursor in acrylate production. Approximately 50% of AA is esterified to form acrylates, while the remaining 50% is purified to 98–99.5% glacial AA for direct industrial applications [13]. MAA and MMA are produced from acetone and sodium cyanide, to form acetone cyanohydrin which is treated with sulfuric acid to form methacrylamide sulfate. This intermediate is hydrolyzed to obtain MAA or esterified with methanol to form MMA [14].

Currently, the majority of the monomers mentioned above are derived from non-renewable feedstocks, primarily natural gas and petroleum (Figure 1). However, the rising demand for waterborne acrylic latexes, coupled with increased awareness of the environmental impact of fossil-based monomers, fluctuating oil prices, and the limited petroleum resources, have driven a growing trend towards the use of renewable sources and bio-based monomers. Although a variety of alcohols (that can be used for esterification of AA and MAA) can be produced from renewable sources, bio-based synthesis of AA remains a challenge, representing a limitation to the production of fully bio-based acrylic monomers [15]. Nonetheless, progress is being made in developing bio-based AA from biomass-derived intermediates such as glycerol, lactic acid, and 3-hydroxypropionic acid [16]. Similarly, promising routes for producing methacrylic acid involve the decarboxylation of itaconic acid (ItA) or the dehydration of 2-hydroxyisobutyric acid [16].

In addition to (meth)acrylate monomers, a range of vinyl monomers are frequently employed to enhance specific polymer properties. Common comonomers include styrene [17], acrylonitrile [18], vinyl acetate [19], and (meth)acrylamide [20]. These monomers introduce distinct functionalities, such as increased chemical resistance, improved adhesion, and enhanced mechanical strength, thereby broadening the application spectrum of waterborne latexes.

## 3. Dispersed-Phase Polymerization

Waterborne acrylic latexes can be obtained through polymerization of acrylic monomers in dispersed media, generally using water as the continuous phase. This process offers a significant advantage over bulk and solution polymerization from an industrial perspective [21]. First, by carrying out the process in water as the continuous phase, the use of hazardous, flammable, and volatile solvents is avoided, making the process safer. Second, the viscosities of the dispersion are also typically lower compared to those of bulk and solvent polymerization, facilitating agitation/homogenization of the system and ensuring efficient heat transfer. In certain cases, such as that of the widely used emulsion polymerization process, polymerization in dispersed media also enables the formation of high-molecular-weight polymers due to compartmentalization and allows for tailoring the size and morphology of the particles. Finally, the resulting polymer particles can be easily isolated and used directly as a colloidal dispersion or latex, whose water-based composition makes it safer to handle.

Dispersed-phase polymerization includes different types of processes that vary in certain mechanistic aspects, such as suspension polymerization, microsuspension polymerization, microemulsion polymerization, emulsion polymerization, and miniemulsion polymerization (MEP) [22]. Here, emulsion polymerization and miniemulsion polymerization polymerization will be explained in more detail due to their higher relevance with respect to previous work that has incorporated biopolymers.

### 3.1. Emulsion Polymerization

A typical emulsion polymerization process consists of an oil phase containing the monomers and an aqueous solution that contains emulsifiers (which can be anionic, cationic, or non-ionic surfactants), and a radical initiator [23].

**Figure 2:**
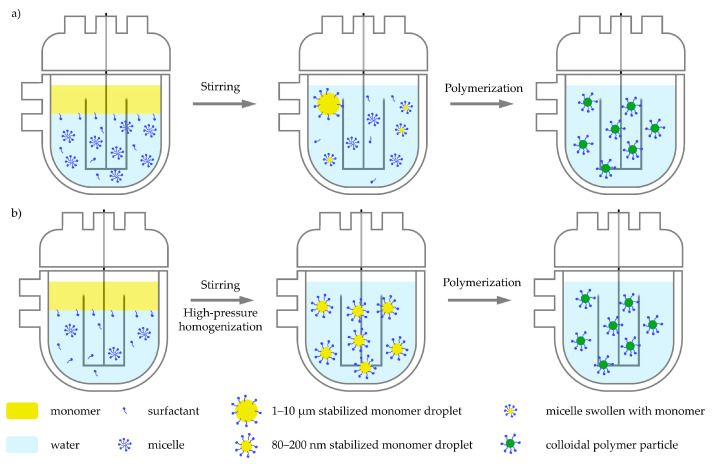
Schematic representation of (**a**) emulsion polymerization and (**b**) miniemulsion polymerization. The first step represents the initial conditions, where two different phases are present before agitation. Dispersed systems are formed and stabilized by surfactant with simple stirring for emulsion polymerization, which generate large monomer droplets and micelles swollen monomer, and strong shear forces, which produce smaller monomer droplets for miniemulsion polymerization. After polymerization, colloidal polymer particles form the latex in both procedures.

Mechanical stirring is applied to break the monomer phase into small droplets (1–10 µm), dispersing them within the continuous aqueous phase (Figure 2a). When the surfactant concentration in the aqueous phase exceeds the critical micelle concentration, micelles can form. Once an initiator is introduced, free radicals are generated in the aqueous phase, triggering the polymerization reaction. For the case of an emulsion polymerization conducted in batch, three distinct stages, commonly categorized as Interval I, II, and III [12], are typically observed as discussed below.

Interval I is closely related to the nucleation process, in which polymer particles begin to form. This interval continues until the particle concentration reaches a point where the formation of new particles becomes unlikely. This point marks the end of the first interval, which generally corresponds to a monomer conversion of approximately 0 to 5%. The mechanisms responsible for the formation of these initial particles can occur via two processes known as homogeneous and heterogeneous nucleation. In homogeneous nucleation, monomers dissolved in water react with a free radical (originating from thermal cleavage of the initiator). Polymerization initially takes place in the aqueous phase until the growing oligomeric radical reaches a degree of polymerization at which point it becomes insoluble. At this point, the particle can precipitate and adsorb surfactant molecules, allowing it to remain colloidally stable. The presence of charges on the polymer chain originating from the initiator fragment can also aid stabilization in this case. Heterogeneous nucleation follows a similar initial pathway, beginning with the formation of an oligomeric radical in the aqueous phase. However, in this case, the oligomer enters a micelle swollen with the monomer, where polymerization continues. In emulsion polymerization of acrylic monomers, heterogenous nucleation is normally the dominant nucleation mechanism.

During Interval II, polymerization proceeds at a constant rate. The polymer particles, which are swollen with the monomer, act as the primary loci of the reaction. This monomer swelling is facilitated by a mass transfer process, in which monomer molecules diffuse from the monomer droplets into the aqueous phase and subsequently into the particles. This interval concludes when the monomer droplets are completely depleted.

In Interval III, which begins at approximately 40% conversion, the polymerization rate starts to decrease. This decreased rate of polymerization is attributed to the decreasing monomer concentration in the polymer particles. At the latter stages of the polymerization, the high polymer concentration can result in an increase in viscosity, which leads to a decrease in the rate of termination and consequentially a higher rate of polymerization. At the end of the reaction, stable colloidal polymer particles are formed with a size on the order of 100 nm.

### 3.2. Miniemulsion Polymerization

Miniemulsion polymerization exhibits differences compared to conventional emulsion polymerization. In this system, the size of the monomer droplets is significantly reduced through mechanical shear forces, typically using sonicators, high-pressure homogenizers, or rotor-stator systems [24]. This process generates droplets with submicron dimensions, typically ranging from 80 to 200 nm. It should be mentioned, that the addition of co-stabilizer is required to prevent diffusion between droplets, preventing Ostwald ripening [25,26]. As a result of the small droplet size, the surface area and number of monomer droplets is significantly higher than a conventional emulsion polymerization. Consequently, the mechanism of particle formation is different in this type of polymerization: rather than occurring within micelles (heterogeneous nucleation), polymerization is initiated when a radical enters a monomer droplet (droplet nucleation), where it propagates [27] (Figure 2b). This approach overcomes one of the key limitations of conventional emulsion polymerization, the necessity for monomer diffusion through the aqueous phase, which can be particularly restrictive for monomers with low water solubility. For a more detailed description of the miniemulsion process, the reader is directed to a number of reviews that describe reaction kinetics [28], practical approaches to perform miniemulsion polymerization [29], and the industrial potential of this process [30].

### 3.3. Pickering Emulsion Polymerization

**Figure 3:**
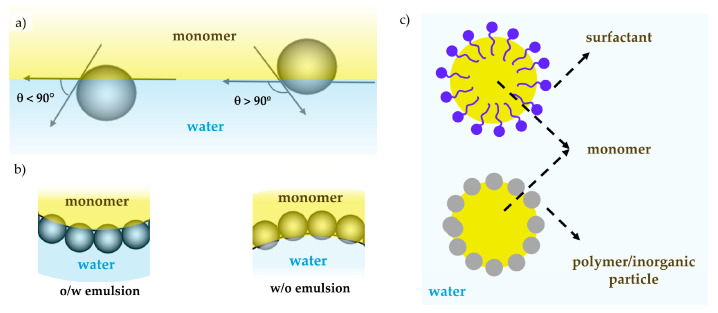
(**a**) Wettability effect of a solid particle at the oil–water interface. The contact angle (θ), measured through the aqueous phase, is shown in two scenarios: less than 90° (left), and greater than 90° (right). (**b**) Corresponding Pickering emulsions: when θ < 90°, an oil-in-water (o/w) emulsion can be formed (left), whereas when θ > 90°, a water-in-oil (w/o) emulsion would be formed (right). Reprinted and adapted with permission from reference [40]. Copyright © 2002 Elsevier. (**c**) Comparison between o/w droplet stabilized by surfactant and by solid particles.

Pickering emulsion polymerization (PEP) is an alternative polymerization process that differs from conventional emulsion and miniemulsion polymerization by replacing traditional surfactants with solid organic or inorganic particles [31,32,33,34,35]. These particles adsorb to the monomer droplet interface, stabilizing the droplets and preventing coalescence. The wettability of the solid particles determines their affinity for the oil–water interface, with an optimal contact angle near 90°, ensuring effective droplet stabilization (Figure 3) [36]. It should be mentioned that unlike the highly dynamic adsorption/desorption process of common surfactants [37], the energy necessary to desorb these particles is various orders of magnitude higher than the thermal energy of the system, often making it an irreversible process [38,39]. At the end of the process, colloidally stable polymer particles are formed, with a shell of stabilizing particles remaining adsorbed at the surface.

### 3.4. Film Formation

**Figure 4:**
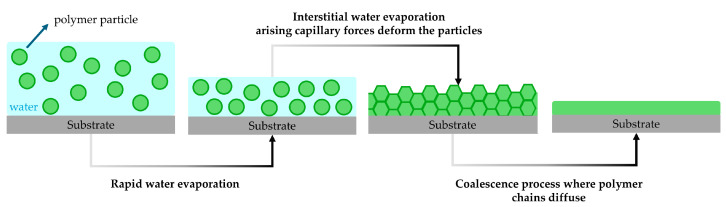
Schematic steps of film-forming process of waterborne acrylic latexes.

Polymer films can be directly cast from waterborne acrylic latexes by evaporating the aqueous phase. This process unfolds through a series of distinct steps involving the drying, deformation, and coalescence of particles (Figure 4) [41,42].

Initially, in the drying process, water evaporates rapidly, causing the polymer particles to move closer together. Later, in the deformation stage, as the interstitial water gradually evaporates, capillary forces arise which compress and deform the particles, effectively filling the remaining air spaces. Finally, the polymer chains within the particles diffuse, merging the deformed particles and eliminating their boundaries. This coalescence process results in a continuous, uniform polymer phase [43,44].

Both the deformation and coalescence stages are significantly influenced by the T_g_ of the polymer. If the T_g_ of the polymer is higher than the temperature at which the drying process occurs, the capillary forces are not sufficient to deform the particles. This leads to stress build-up and cracking during the drying of the film [45]. A similar limitation arises during coalescence. If the process temperature is below the T_g_, the polymer chains will lack the required mobility for effective interdiffusion, hindering the elimination of boundaries between particles and preventing the formation of a uniform, continuous phase. Polymers with a low T_g_, often referred to as “soft” polymers, can form continuous films at lower temperatures, whereas “hard” polymers with a higher T_g_ require elevated temperatures to achieve coalescence. The parameter that defines the ability to form a film is the minimum film formation temperature (MFFT) which is the lowest temperature at which a film can form a continuous layer without cracks and defects.

## 4. Biopolymer–Acrylic Hybrids

Biopolymers are polymers that are produced by or derived from living organisms, such as plants or bacteria [46]. It may be noted that these are distinct from bio-based polymers, which are those polymers at least partially made from renewable sources. The growing demand for environmentally sustainable materials and the reduction of reliance on fossil fuels have driven extensive research into alternatives to petroleum-based materials. As a consequence, biopolymers have emerged as promising candidates due to their biodegradability, biocompatibility and their mechanical, barrier, thermal, and optical properties [47]. Additionally, they can typically be obtained by eco-friendly methods, contributing to reduced environmental impact. Among the most relevant biopolymers are proteins including casein, whey, collagen and soy; and polysaccharides, such as cellulose, starch, chitin, xanthan gum, and alginate. This collection of biopolymers offers a diverse range of functional properties that make them attractive for various applications.

**Figure 5:**
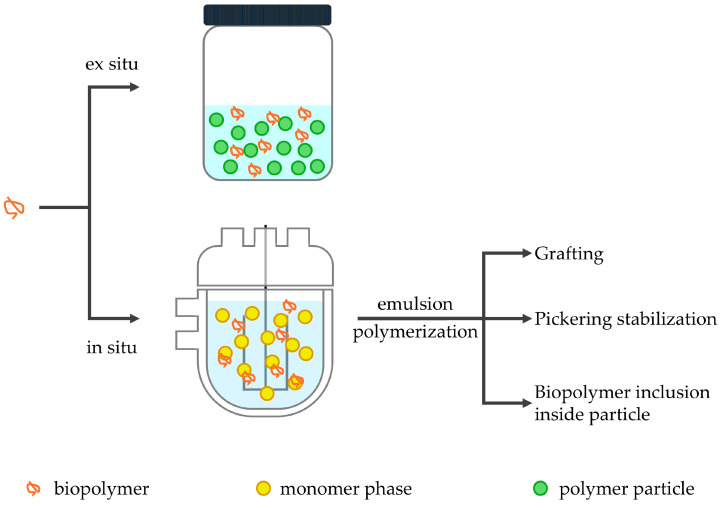
Simplified representation of approaches for biopolymer addition into acrylic latexes. Top: ex situ addition where biopolymer is added on preformed latex. Bottom: in situ addition, where biopolymer is added before the polymerization.

The desire to exploit these biopolymers in acrylic latex systems has led to a significant effort from researchers to find optimal ways to combine the two materials. The resulting hybrid materials are expected to show a combined and synergistic improvement of a series of properties in order to meet the requirements of the most common applications of these latexes. To achieve this integration, various approaches have been explored. The simplest way is ex situ incorporation, in which the biopolymer is physically mixed with a pre-synthesized acrylic latex. An alternative to this is the in situ approach, in which the biopolymer is present in the polymerization media (Figure 5).

In situ approaches promote the direct grafting of acrylic polymers onto the biopolymer backbone, as is explained in more detail below. In those cases, some parameters, related to grafting, must be evaluated; such as grafting efficiency (GE), and the percentage of grafting (PG) which can be defined as follows:

(1) $$\mathrm{Grafting}\mathrm{efficiency}\left(\mathrm{GE}\right)=\frac{\mathrm{wt}.\;\mathrm{of}\;\mathrm{grafted}\;\mathrm{synthetic}\;\mathrm{polymer}}{\mathrm{wt}.\;\mathrm{of}\;\mathrm{total}\;\mathrm{synthetic}\;\mathrm{polymer}}\times 100$$

(2) $$\mathrm{Percentage}\;\mathrm{of}\;\mathrm{grafting}\;\left(\mathrm{PG}\right)=\frac{\mathrm{wt}.\;\mathrm{of}\;\mathrm{grafted}\;\mathrm{synthetic}\;\mathrm{polymer}}{\mathrm{wt}.\;\mathrm{of}\;\mathrm{biopolymer}}\times 100$$

As will be discussed, researchers have targeted the development of novel hybrid acrylic latexes that not only maintain the advantages of synthetic polymers but also introduce biodegradability, enhanced properties, and improved sustainability, making them viable alternatives for conventional formulations. In the following sections, the main approaches for incorporation of proteins and polysaccharides into acrylic latex systems will be detailed.

### 4.1. Proteins

Proteins are natural polymers composed of repeating units called amino acids, linked together through peptide bonds. These biopolymers can be derived from either animal or plant sources. The specific sequence and type of amino acids determine the structure and properties of the resulting protein, leading to a vast spectrum of biopolymers within the protein family. Based on their length, shorter chains of amino acids are referred to as peptides, while longer, more complex macromolecules are classified as proteins. Proteins often fold into intricate three-dimensional structures, which are critical to their function. Additionally, proteins can associate with other protein molecules to form larger, more complex assemblies.

The chemical diversity among amino acids results in a wide range of functional groups within proteins, offering numerous possibilities for chemical modification or for grafting synthetic polymers onto the protein backbone [48]. However, in their native folded state, many of these functional groups can be hidden within the protein’s interior, limiting their accessibility. To expose these reactive sites, proteins often undergo denaturation, a process that unfolds their three-dimensional structure, making previously inaccessible functional groups available for further chemical reactions or modifications [49].

Proteins are primarily produced and incorporated into food and beverage products to increase their nutritional content, improve texture, enhance stability, and enhance overall product quality [50,51]. However, the use of proteins as additives for industrial applications is rising, particularly for adhesives, paints, and coating formulations [52].

It is important to recognize that practical use of biopolymers in commercial acrylic systems is dependent on their widespread availability. In the case of casein, production was around 427.9 kilotons in 2024 and is expected to increase up to 581.7 kilotons by 2033 [53], with New Zealand, Ireland, France, and the Netherlands being the top exporters [54]. Annual production of gelatin was accounted to be 464 kilotons in 2024 [55]. In 2023, the total world production of soybean was around 371.17 million tons, with Brazil, the United States, and Argentina being the main producers [56]. However, not all soybeans are processed into soy protein; a significant portion is used for animal feed and oil extraction. Industrial obtained soy protein is divided into three products as a function of the protein concentration, soy flour (>50%), soy concentrate (>65%), and soy protein isolate (SPI) (>90%). The production volume of SPI (around 4.2 million tons in 2023 [57]) is higher than casein, which is explained by the broader cultivation and utilization of soybean worldwide. In contrast, casein is inherently limited by milk production. This also has an environmental impact. SPI has a significantly lower environmental impact than casein, with reduced carbon emissions, water use, and land requirements. SPI’s plant-based origin and efficient soybean cultivation make it more sustainable, while casein’s dairy-based production leads to higher greenhouse gas emissions, greater water usage, and increased land use [58].

The use of proteins for the synthesis of hybrid nanoparticles, primarily obtained through emulsion polymerization and miniemulsion polymerization, was reviewed in 2021 by Cencha and coworkers [59]. Building on that work, here, an overview of the topic will be presented followed by a review of the latest research on combinations with waterborne acrylic latexes. Among the reviewed proteins, we have focused on casein, soy protein, and gelatin, as they have been more extensively reported. Regarding whey protein, limited publications address its use in waterborne acrylic latexes. Regarding the highly hydrophobic zein protein, since it is typically combined with other biopolymers (e.g., casein), it will be discussed in the relevant sections.

#### 4.1.1. Casein

Caseins are a series of phosphoproteins that constitute around 80 wt% of the total protein content in milk, with whey protein making up the remaining 20 wt% [60]. It exists as a caseinate–calcium–phosphate complex. These proteins have been used for food applications, paper, textile, for the manufacture of artificial fibers, coatings, adhesives, paints, leather treatment, surfactant, and pharmaceuticals [61].

Casein can be obtained through different processes, including acidification, rennet treatment and microfiltration. The most common is acidification, where the lowering of the pH of the skimmed milk (fat should be firstly separated from milk) promotes calcium displacement from the casein molecules, and the calcium phosphate associated with the complex is converted into soluble ions. At a pH of around 4.6 (the isoelectric point of casein), maximum precipitation occurs. Organic and mineral acids can be used indistinctly, with hydrochloric acid, sulfuric acid, and lactic acid being the most common. The caseins produced by different acids are almost identical. Alternatively, casein coagulation can be promoted by rennet, which is a series of enzymes produced in the stomachs of ruminant mammals [62]. After obtaining precipitated casein either by acidification or rennet treatment, the obtained casein undergoes several procedures which include separation, washing, dewatering, drying, tempering, grinding, grading, blending, and bagging [61]. Caseinates, which are the salt form of acid casein, can be obtained during this process by reacting dry acid casein with dilute alkali solutions [63].

Microfiltration is an alternative methodology to obtain casein whose product has been categorized as micellar casein concentrate (MCC). This procedure is the cleanest methodology which is mainly applied for use in the food industry [64,65]. It should be noted that industrial casein (that is not aimed at being used in the food industry) is cheaper to produce due to the reduced restrictions.

**Figure 6:**
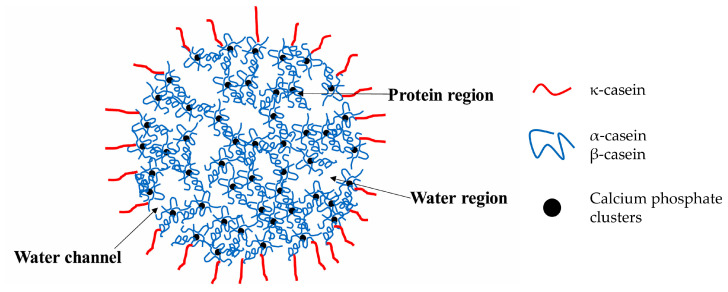
Schematic illustration of casein micelles structure: black spheres represent the calcium phosphate nanoclusters; blue coils represent αS and β-caseins which can combine with nanoclusters; red lines present on the outermost part of the surface represent κ-caseins which provide steric and electrostatic stabilization. Reprinted and adapted with permission from reference [69]. Copyright © 2022 Elsevier.

From a structural point of view, caseins present four distinct structures in different proportions: α_S1_ (40%), β (35%), κ (15%), and α_S2_ (10%) [66]. Each type of casein exhibits unique physicochemical properties due to its composition [67]. It is assumed that these structures remain aggregated, forming micelles containing bivalent cations (Figure 6), but the exact structure is under discussion [68].

Obtaining casein through acidic precipitation is a simple and cost-effective process, making casein salts an affordable material [61]. However, isolating the individual casein structures requires more sophisticated and specialized procedures, as reported for α_S_-casein [70,71], and κ-casein [71]. Further research has been carried out to isolate β-casein [72,73], which is particularly interesting due to its critical micelle concentration, which ranges from 0.5 mg/mL to around 2 mg/mL, depending on temperature, ionic pressure, and pH. These micelles typically consist of 15–60 monomers with a radius ranging from 8–17 nm [66].

Reactive sites in casein include hydroxyl and phenols groups (from tyrosine, serine, and threonine), amino groups (from lysine), and carboxylic acid groups (from aspartic and glutamic acids). Viora and coworkers reviewed all options that casein protein has to be functionalized with different bio and petroleum-based compounds [48]. They illustrated the possibility to incorporate sugars, crosslink casein chains, and the most interesting with respect to the present review, the ability to react with vinyl monomers such as acrylonitrile, methacrylate, and acrylamide.

The emulsifying properties of casein have made it an excellent biobased compound to use in acrylic latex systems. This allows for the synthesis of hybrid casein–acrylic latexes by emulsifier-free emulsion polymerization (EF-EP). The resultant hybrid films are composed of a percentage of biobased polymer that combine the properties of the casein and the synthetic polymer.

##### Ex Situ Addition

There are only limited reports on the ex situ addition of casein, as most research has primarily focused on the emulsifying properties of casein. Qiang et al. added an aqueous solution of casein into an acrylic latex to use it as a crosslinking agent [74]. The crosslinking reaction occurred between the primary amino group in the casein backbone and the ketone groups of acetoacetoxyethyl methacrylate (AAEM) present in the acrylic polymer. The crosslink density was adjusted by varying the AAEM concentration; as the concentration increased, hardness, solvent resistance, and tensile strength improved, while elongation at break decreased.

##### In Situ Addition

As previously stated, proteins, including casein, can stabilize o/w emulsions by creating a repulsive barrier around the oil droplets. This stabilization arises from the combination of electrostatic and steric repulsions generated by the protein layer adsorbed onto the droplet surface. As a result, an increase in the effective thickness of the adsorbed protein layer can greatly improve the stability of an emulsion. In this line of work, Liu and Damodaran modified β-casein to acquire a branched morphology with increased steric repulsive interactions [75]. Sitohy et al. esterified β-casein with different alcohols to improve the emulsifying properties at acidic pH [76]. Although no polymerization in dispersed media was reported in those studies, it may be concluded that the improvement of the colloidal system will enhance the shelf-life of the acrylic polymer latexes, avoiding coagulation/flocculation process.

Different strategies have been used to polymerize acrylic monomers in the presence of casein as the emulsifier as detailed below. They differ on the use of native or modified casein and the initiator system used, which are closely related to the extent to which grafting occurs.

##### Use of Radical Initiators

The most common initiators used in emulsion polymerization, persulfates, can be utilized for grafting casein to obtain a stable latex with a core–shell structure, where the acrylic phase forms the core and the casein phase forms the shell. Persulfate radicals can abstract hydrogen atoms from the protein backbone, generating radicals that react with vinyl bonds in acrylic monomers, thereby grafting them onto casein [59]. For example, this type of synthesis was carried out by Mohan and coworkers. They studied the graft-polymerization of butyl acrylate (BA) [77], *n*-butyl methacrylate (BMA) [78], and methyl acrylate (MA) [79] with casein, initiated by potassium persulfate (KPS) in aqueous media. Generally, despite differences in reactivity depending on the monomer used, increasing monomer concentration led to higher polymerization rates, graft copolymerization rates, and PG, while GE tended to plateau or decrease. The initiator concentration, casein concentration, and reaction temperature exhibited a parabolic effect on the grafting parameters, initially enhancing all values with a subsequent stabilization or decrease. This trend was primarily attributed to the termination of primary radicals. In addition to the thermal cleavage for radical generation offered by persulfates, radicals can also be produced via redox systems, as demonstrated for MA grafting onto casein using the KPS/ascorbic acid redox pair [80].

##### Redox Pair with Amino Group from Casein

**Figure 7:**
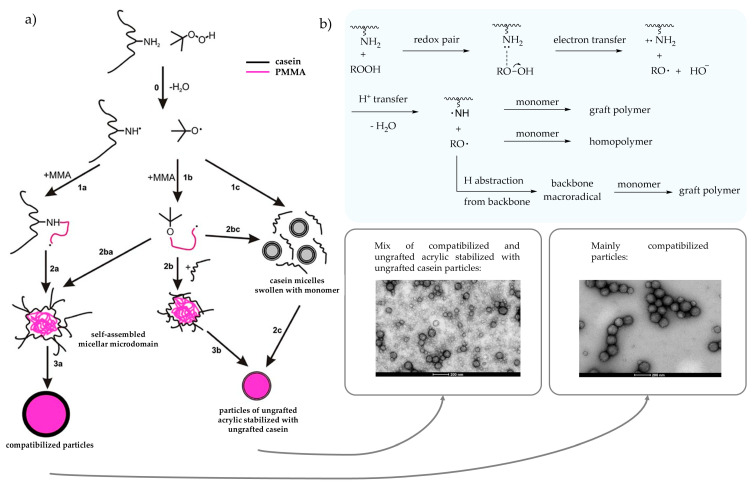
Schematic representation of the two paths for the formation of amphiphilic core–shell nanoparticles and their corresponding SEM micrographs, adapted from [83]. (**a**) Redox radical generation, (1a) amino radical initiation, (1b) *tert*-butoxy radical initiation, (1c) *tert*-butoxy radical entering in casein micelles swollen with monomer, (2a) self-association of casein-g-polyacrylic, (2ba) oligomer entering in self-assembled micellar microdomain, (2b) oligomer stabilized by casein, (2bc) oligomer entering in casein micelles swollen with monomer, (3a) formation of compatibilized particle, (3b) and (2c) formation of polyacrylic particles stabilized with ungrafted casein. Reprinted and adapted with permission from reference [83]. Copyright © 2014 John Wiley and Sons. (**b**) Proposed mechanism for the graft copolymerization of MMA from water-soluble polymers containing amino groups, adapted from [82]. Reprinted and adapted with permission from reference [82]. Copyright © 2002 American Chemical Society.

Zhu and Li reported the possibility of grafting MMA onto soluble polymers containing amino groups (i.e., casein) by *tert*-butyl hydroperoxide (tBHP), which forms a redox pair with amino groups to generate an amino and tertbutoxy radical that can react with the vinyl group of MMA (Figure 7b) [81,82]. It should be noted that the resulting particle had a core–shell morphology where amphiphilic casein (shell) surrounded the acrylic phase (core). A deeper study was reported specifically for casein protein, where they analyzed how synthetic parameters affected conversion, PG, GE, stability, and particle size. A higher GE was achieved using a more water soluble initiator, tBHP compared to cumene hydroperoxide (CHP). Additionally, the GE was also higher when the tBHP/amino-casein redox pair was used instead of pure persulfate grafting, also decreasing the oxidative degradation of the protein. Increasing the monomer concentration was found to increase particle size and PG, while the GE plateaued. The study also revealed that a maximum solid content of 31 wt% could be achieved (with a casein-to-MMA weight ratio of 1:4) without significantly broadening the particle size distribution. Further work was reported by Picchio et al. who studied the emulsion polymerization of MMA at high casein concentrations. They revealed that at low casein concentrations, mainly grafted casein-g-acrylic was produced (i.e., compatibilized particles). At high casein concentrations, secondary nucleation of non-grafted casein–acrylic particles was promoted (i.e., uncompatibilized particles) Figure 7a [83,84]. The presence of free (ungrafted casein) plays a critical role on the final properties of the hybrid polymer, as explained below.

##### Casein–Caprolactam

**Figure 8:**
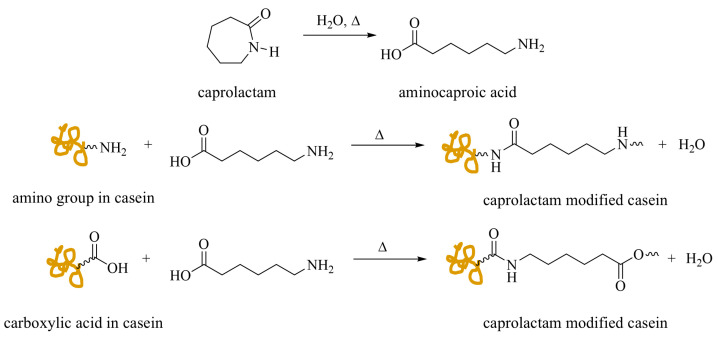
Polycondensation reaction mechanism of caprolactam initiated by casein backbone moieties. Reprinted and adapted with permission from reference [89]. Copyright © 2012 Elsevier.

Ma and coworkers reported a series of studies on a feasible casein modification method to enhance colloidal stability for the synthesis of hybrid acrylic polymers via emulsifier-free emulsion polymerization [85,86,87,88]. The functionalization of casein was carried out in an alkaline aqueous solution using triethanolamine, where casein was dissolved and heated at 65 °C for 2 h. Subsequently, the mixture was heated to 75 °C, and a caprolactam solution was added dropwise, reacting for an additional 2 h. Typically, the casein load was 2.3 to 7.0 times the weight of caprolactam, while the caprolactam solution had a concentration ranging from 10 to 25 wt%. The caprolactam underwent condensation polymerization, initiated by the amine (-NH_2_) and carboxylic acid (-COOH) groups of casein (Figure 8). The resulting casein–caprolactam (CPL-casein) dispersion could be utilized for emulsifier-free emulsion polymerization by incorporating acrylic monomers into the dispersion and stirring the mixture for an additional 0.5 h before introducing the initiators. The initiators reported included persulfates (KPS and ammonium persulfate (APS)) for thermal initiation, as well as redox systems (KPS or APS paired with NaHSO_3_). Among these, the KPS/NaHSO_3_ redox pair demonstrated higher values of PG and GE [86]. While the solid content of the emulsion polymerization was not explicitly mentioned in the articles, it can be inferred to range from 7 to 20 wt%, based on the reported data.

##### Methacrylated Casein

Controlling the GE is essential to tailor the final latex properties. Picchio et al. achieved this by introducing vinyl moieties onto casein through its reaction with glycidyl methacrylate (GMA) to obtain a methacrylated casein [90]. The concentration of vinyl moieties could be adjusted by varying the GMA-to-casein ratio, with a maximum functionality reported of 32 vinyl double bonds per molecule of casein [90]. To synthesize this casein derivative, casein was dissolved in alkaline water (pH higher than 10) at 50 °C, followed by the addition of GMA and left to react for 4 h. Since the reaction media was in water, the resulting solution could be directly used in emulsion polymerization, offering a significant advantage. Different degrees of functionalization were evaluated, showing that while higher vinyl concentrations increased the compatibilization between casein and the acrylic phase, they also reduced the overall monomer conversion. This decrease was attributed to the reduced availability of active amine groups (primary amines) on the casein backbone, which initiates the polymerization. The enhanced compatibility between casein and the acrylic phase was clearly evidenced by the homogeneous distribution of both phases through atomic force microscopy (AFM) images. Methacrylated casein was exploited by later works to produce hybrid latexes [91,92,93].

##### Properties and Applications

Regarding the applications of casein in potential commercial and industrial products, as discussed above, it can act as an emulsifier, enabling emulsion polymerization to be performed without the need for additional surfactants. However, its role extends beyond its emulsifying effect. Casein can function as an additive to fine-tune certain properties of the final film. The primary applications of these latexes are in adhesives, paints, and coatings. All of these applications require a low MFFT, which has been achieved by using BA and 2EHA as soft monomers. Notably, casein did not negatively affect the film formation of hybrid films. Despite its high T_g_ of 180 °C, its MFFT is relatively low (11 °C) due to its high level of hydration, with water acting as a plasticizer [84]. Differences in surface roughness have been reported; generally, higher casein loads led to smoother surfaces and more transparent substrates [84,94]. Moreover, this hybrid system provides longer open times (the period during which the applied coating can be modified without causing imperfections in the dried film). This behavior is directly associated with the presence of casein, which acts as hydroplasticizer, retaining water for extended periods [90]. The presence of casein increases the cohesiveness of the resulting hybrid material, enhancing tensile strength, while reducing elongation at break as casein concentration increases [84]. As mentioned, the ratio of compatibilized to uncompatibilized components affects mechanical properties due to the greater number of acrylic chains covalently bonded to casein. Consequently, a synergistic effect was observed in methacrylated casein-g-BA/MMA, where both tensile strength and elongation at break increased [90]. As expected, the incorporation of casein made the hybrid latex more biodegradable compared to pure acrylic films [84,90,92,94].

Cencha et al. reported a crosslinked system based on the Michael addition or Schiff-base reaction between tannic acid (TA) and the amino groups of casein [92]. TA was dissolved in a 1M NaOH solution and added to a methacrylate casein-g-acrylic latex. The authors observed that dried films were able to crosslink at room temperature, but the complete process took a few days. The intensity of the brownish color of the film increased as TA concentration increased, making it more opaque. However, the addition of 5 wt% TA imparted UV-blocking properties of the film, which are typically achieved using petroleum-based organic compounds. Higher TA concentrations also led to reduced water uptake. The final cross-linked matrix did not significantly affect the biodegradability of the hybrid film. Incorporating crosslinking agents into latexes promoted the coagulation of particles, increasing instability, which led to increased viscosity and particle size of the latex.

Some composite systems have been reported for incorporating nanomaterials into casein–acrylic hybrid latex. For example, Xu et al. reported a core–shell silica nanocomposite prepared via in situ emulsion polymerization [85]. Regarding the final structure, caprolactam–casein acted as a colloidal stabilizer during silica formation and acrylic polymerization until an inorganic layer formed around the particle, creating an organic core composed of CPL–casein–polyacrylate and an inorganic silica shell. The in situ formation of latex ensured the uniform dispersion of silica compared to the direct addition of silica into latex, thereby improving compatibility between all elements. Compared to CPL–casein–polyacrylate films, silica-containing latexes exhibited enhanced thermostability, higher hydrophobicity, lower water absorption, improved tensile strength, and reduced flexibility.

Casein has also been used as a compatibilizer between different phases in composite materials, as reported by Liu and coworkers [95]. They prepared a composite via latex compounding, mixing natural rubber latex with a casein–acrylic latex and a standard acrylic latex for comparison. They observed that casein enhanced the compatibility between the rubber and the acrylic phase composed of MMA/BMA. Improved compatibility led to the formation of a smoother surface and better mechanical properties compared to those of the casein-free formulation.

An important feature of the hybrids discussed above is that the incorporation of casein can reduce the amount of petroleum-based content in the final latexes. Following this direction, Allasia and coworkers studied the use of bio-based acrylic monomers in emulsifier-free emulsion polymerization, stabilized by methacrylated casein with the objective of maximizing the bio-based content of the final latexes [91]. Low conversions were obtained when pure bio-based monomers (isobornyl methacrylate and 2-octyl acrylate) were polymerized, which was attributed to their low solubility in water. To address this issue, petroleum-based MMA was added to increase the concentration of vinyl monomers in the water phase, thereby promoting polymerization between methacrylate–casein and bio-based acrylates. Once again, casein functionalization led to improved compatibilization between both phases (protein and acrylate), enhancing properties such as swelling resistance and water resistance. A slightly improved hydrophobic surface was observed with higher bio-based acrylate content, along with a slight improvement in biodegradability.

Further work by the same authors addressed the challenge of obtaining percolated structures derived from the coalescence of core (acrylic)–shell (casein) particles. This system resulted in higher susceptibility to water due to the exposure of the highly hydrophilic casein phase. To mitigate this issue, an inverse structure was synthesized, in which the protein primarily remained in the core phase. This was achieved through seeded batch and semi-batch emulsion polymerization using a casein/zein bioparticle as seed [96]. These bioparticles were synthetized through an anti-solvent method, obtaining highly hydrophobic zein particles dispersed in water thanks to the casein stabilization. After the emulsion polymerization a multi-lobular core–shell structure was obtained where the core was composed of casein/zein protein, while the shell was made of a BA/MMA copolymer. Due to this novel approach, various parameters, including the synthesis method, protein/acrylate ratio, and zein/casein ratio, were investigated. Each parameter exhibited an optimal value, as any deviation resulted in negative effects on stability, compatibility, or conversion. It was found that semi-batch emulsion polymerization was superior to batch polymerization, yielding more stable latexes. Moreover, feed rate was identified as a key parameter for stabilization. Brownish films were obtained due to the presence of zein protein. The best results in terms of latex stability and compatibility were found for protein/acrylic ratios of 20/67–20/50. Additionally, the concentration of casein in the zein/casein seed had a significant impact, as excess free casein in the aqueous phase promoted secondary nucleation. Table 1 provides a summary of the various methods for preparation of casein-based acrylic dispersions and the proposed applications.

#### 4.1.2. Soy Protein

The raw material to obtain soy protein is the soybean, which contains approximately 36% protein, 15% soluble carbohydrates, 15% insoluble carbohydrates, and 18% oil [58]. These percentages can vary as a function of the soybean variety. SPI is the most concentrated form of commercially available soybean protein and contains a minimum of 90% protein on a dry matter basis [58]. To obtain soy protein, several physicochemical processes should be carried out in order to remove other components. As these steps are completed, more concentrated protein products are obtained: soy flour (minimum of 50%),and soy concentrates (>65%) [58].

Soy proteins consist of a mixture of albumins and globulins, of which 90% are storage proteins that exhibit a globular structure. These storage proteins primarily include 7S (β-conglycinin) and 11S (glycinin) globulins. In globulins, the protein subunits are held together through hydrophobic interactions and hydrogen bonds [97]. SPI solubility in water is influenced by pH, ionic strength, temperature, and degree of protein denaturation. Solubility improves in alkaline media and decreases with higher ion concentrations, especially for divalent ions. Temperature has a limited effect and protein denaturation decreases its solubility due to the exposure of hydrophobic groups [98].

Regarding the properties of pure SPI, they are considered brittle due to their poor mechanical integrity and low toughness [99]. The addition of plasticizers, while reducing the tensile strength, improves the overall performance of the material [100]. Processing conditions can affect the integrity of the protein, as denaturation can occur. In the case of 7s, this occurs at around 75 °C, while 11S is more stable and is denatured at 225 °C [100]. Related to their thermal properties, soy protein films show a significant weight loss above 200 °C, presenting a limited thermal stability [99]. In terms of barrier properties, the polar nature of the polymeric chain contributes to a reduced oxygen permeability which could be compromised due to their high water absorption [97], which also affects to its adhesive properties [49].

##### Ex Situ Addition

Regarding ex situ approaches to acrylic latex–soy protein systems, Luo et al. mixed soybean meal solution (a mixture mainly composed of proteins, polysaccharides and water) with a synthetic crosslinker before adding a commercial acrylic latex [49]. This direct mixing method led to a high soy content in the hybrid polymer while maintaining a relatively high solid content (up to 36.38%). Alternatively, Wang et al. blended a polyacrylate emulsion with a soy protein solution, both at 10% of solid content, stirring the mixture at 60 °C for 1.5 h, followed by cooling at room temperature and filtering to remove the coagulated fraction [101].

##### In Situ Addition

For in situ polymerization, both unmodified soy protein [97,102] and modified soy protein [102,103,104,105] have been utilized. The modifications of the soy protein were performed to improve the compatibility between the biopolymer and the synthetic polymer. Methods included modification with methacryloyl silane [105], alkaline treatment to increase the availability of crosslinkable groups by disrupting the protein conformation [102,104] or protease treatment [103]. Both emulsion and miniemulsion polymerization processes have been employed for polymerization, using persulfates as initiators, which can promote crosslinking between soy protein and acrylic polymers by generating radicals on the amino and carboxyl groups [104].

##### Properties and Applications

In acrylic–soy protein hybrids, the acrylic component offers enhanced film formation, flexibility, water resistance, and adhesion, while the soy protein enhances the hardness and cohesiveness of the material. Crosslinking agents are commonly used in soy–acrylate formulations to significantly improve the water resistance. Various crosslinkers have been used, including synthetic epoxy-based crosslinkers [49], diphenyl methane diisocyanate [101], and 4-hydroxybenzophenone (UV curing reagent) [103]. Generally, increasing the crosslinking degree of hybrid latexes improves thermal stability [49,97], and increases tensile strength while reducing elongation at break [103].

Further functionalization of hybrid soy protein-acrylic systems was reported by Su et al. who introduced 4-hydroxybenzophenone (HBP) as a UV curing agent. Under UV light exposure, benzophenone initiates a UV curing process that can aid further polymerization and crosslinking. They compared the differences between ex situ and in situ addition of both SPI and HBP. Results showed that faster photolysis, higher curing efficiency and enhanced mechanical properties were obtained from the in situ addition.

Hybrid latexes have been used for adhesives and coatings for wood, due to the traditional applications of soy protein, which can be improved (mainly for its water resistance) by the incorporation of polyacrylates. As a result, soy protein can be added into acrylic latexes to increase the bio-content of the final material while keeping the values required for the end-use application. Kisku at al. reported the improved oxygen barrier properties for films composed of PMMA and soy protein (up to 5 wt%), obtained via hot pressing of the precipitated latex. The resulting barrier properties make the material suitable for packaging materials [97]. Table 2 provides a summary of the various methods for preparation of soy protein based acrylic dispersions and the proposed applications.

#### 4.1.3. Collagen

Collagen is a structural protein in animals, constituting approximately 30% of total protein content [106]. It includes a series of proteins that share a common characteristic: their arrangement in a triple helix of three polypeptide chains. However, their size, function, and tissue distribution can vary [107]. Collagens are classified based on their supramolecular organization, with fibril-forming collagens being the most common type, representing 90% of total collagen. These collagens are primarily found in tissues such as bones, dermis, ligaments, cornea, cartilage, and skin [107]. The principal sources of native collagen are bovine and porcine connective tissues. However, due to increasing demand, alternative sources such as fish tissues and byproducts, as well as chicken, duck, and rabbit skins, have begun to be used [108].

Two main products are derived from collagen: gelatin and hydrolyzed collagen (HC). Gelatin is produced through the partial hydrolysis of collagen under either acidic or alkaline conditions, with molecular weights ranging from 15 to 250 kDa [109]. Due to its amphiphilic nature, gelatin exhibits emulsifying properties, which can stabilize o/w dispersions. However, its effectiveness depends on the gelatin source [89].

Hydrolyzed collagen consists of low-molecular-weight peptides, typically ranging from 3 to 6 kDa [108]. It can be obtained through chemical, enzymatic hydrolysis, or through a combination of both processes. In this approach, gelatin is first produced, as described earlier, and then subjected to further enzymatic treatment to obtain HC [108]. Unlike gelatin, HC is water soluble and does not gel. Moreover, it does not have the film formation properties of collagen and gelatin [110].

##### Ex Situ Addition

As previously discussed in the case of casein, Qiang et al. used gelatin as a crosslinking agent, which reacted with AAEM present in an acrylic phase [74]. The resulting films containing gelatin were more opaque than those with casein, which was undesirable. Additionally, the tensile strength values of the gelatin-containing films were lower than those of casein-based films.

##### In Situ Addition

Direct grafting in emulsifier-free emulsion polymerization with persulfates, both APS and KPS, has been reported for crosslinked gelatin [111], collagen with different degrees of hydrolysis [112], and recovered collagen from leather waste [113]. Several factors, including monomer solubility, initiator concentration, reaction time, and gelatin concentration influenced grafting parameters (PG and GE) [111]. The stability of the emulsion is dependent on the grade of hydrolysis of collagen [112].

A redox system using peroxides and amino groups present on the gelatin backbone was reported by Li and coworkers, as previously mentioned [82]. Studies based on this synthetic approach were reported by Luque et al. who used hydrogen peroxide instead of tBHP to avoid the release of VOCs [114]. Compared to casein, differences in the particle sizes were observed as a result of the lower emulsifying ability of HC. The critical micelle concentration of HC was around 120 mg/mL compared to 0.1 mg/mL for casein, which lead to the formation of larger hybrid particles [114]. Additionally, limited PG was reported, independent of the amount of HC added. Recently, the same authors reported the methacrylation of HC, emulating the methacrylation process reported for casein [115]. The number of methacrylic groups added per HC molecule was 5.9, 3.5, and 1.7, which was less than the reported 32 vinyl groups per molecule of casein [90]. This could be related to the differences in molecular weight of the two biopolymers.

Wang and coworkers developed a gelatin-poly(methacrylic acid) emulsion in which carboxyl groups interacted with amide groups of gelatin via H bonding. This interactions reduced the upper critical solution temperature of gelatin [116], a necessary modification to prevent gelation at common storage temperatures. This effect is usually achieved by degrading gelatin into lower molecular weight fragments, which could compromise the final properties. The resulting emulsion was blended with a poly(butyl acrylate) latex to balance the final properties.

##### Properties and Applications

Among the reviewed works, paper sizing was one of the proposed end-use applications. Collagen presents a high bonding strength with paper fibers, but its performance alone is insufficient, which is why it is grafted with synthetic polymers [112]. Compared to untreated paper, grafted collagen improved tensile strength, tearing strength [112], ring crush index, cobb test, and displayed increased surface hydrophobicity [112,113,116]. Wang et al. obtained a hybrid latex using collagen from leather waste that could be used as a sizing agent for paper, illustrating the potential use of a second life of biopolymers [113]. With regards to the biodegradability, coated paper was completely degraded in 15 days [116].

Luque and coworkers reported the use of grafted gelatin–acrylic latexes for adhesive applications [114,115]. As mentioned, they obtained hybrid latexes through different synthetic approaches; direct grafting using a peroxide redox system with amino group and methracrylated HC (MHC). The synthetic method had a remarkable impact on compatibility, microstructure, and final properties of the films. Both hybrid latexes showed unaffected film-forming capacities; however, films derived from MHC were more transparent than those obtained via redox grafting, indicating better compatibility between both phases. AFM phase images revealed phase segregation on redox grafted hybrids, whereas a more homogeneous distribution was observed for the MHC. Similar thermal behavior was reported for both systems, falling between that of pure HC and acrylic polymers. Regarding mechanical properties, unmodified HC exhibited a higher tensile strength, young modulus, and lower elongation at break. In contrast, adhesive properties were improved for MHC, independent of the degree of functionalization. Moreover, adhesive properties were moisture dependent for both methods. Table 3 provides a summary of the various methods for preparation of collagen based acrylic dispersions and the proposed applications.

### 4.2. Polysaccharides

**Figure 9:**
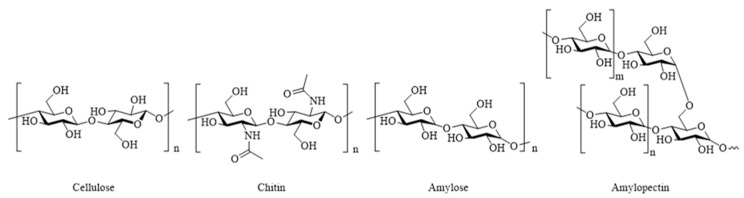
Chemical structure of different polysaccharides.

Polysaccharides are the most abundant natural macromolecular polymers and are derived from a variety of renewable sources including algae, plants, and microorganisms, such as fungi and bacteria. These biopolymers are composed of monosaccharide units covalently bonded by glycosidic linkages and exhibit structures ranging from linear to highly branched. In living organisms, their primary functions are either structural or for energy storage. Structural polysaccharides include cellulose and chitin, which both possess a fibrillar structure. Chemically, cellulose consists of glucose monomers, whereas chitin is composed of *N*-acetylglucosamine (Figure 9). As an energy storage polysaccharide, starch is composed of a variable proportion of amylose (linear) and amylopectin (branched). Although starch and cellulose are composed of glucose units, the linkages differ: starch contains α-(1,4)-glycosidic bonds and α-(1,6)-linkages for amylopectin branches, whereas cellulose and chitin are characterized by β-(1,4)-glycosidic bonds.

The presence of -OH, and -NH(OC)- functional groups allows polysaccharides to form strong hydrogen bonds between polymer chains, contributing to their structural integrity. These polysaccharides can also undergo further functionalization, as will be discussed in later sections, broadening the types of available biopolymers.

Assessing the global production of these biopolymers, cellulose is primarily obtained from plant-based sources, while chitin is extracted mainly from animal sources, principally from exoskeletons of crustaceans.

Cellulose is predominantly derived from wood pulp, with global production reaching approximately 195.79 million tons in 2022 [117], and from cotton linters, contributing an additional 25.2 million tons in 2019 [118]. Cellulose is mainly used as pulp for paper manufacturing. In contrast, its derivatives, such as cellulose fibers, cellulose esters and ethers, and microcrystalline cellulose, find application across a broad range of industries, serving different roles depending on the end use [119].

The global production of starch was estimated at approximately 93 million tons in 2020, with corn accounting for nearly 75% of this volume [120]. While the food industry remains the predominant driver of starch production, the expansion of non-food sectors, particularly in developed economies, is significantly contributing to the rising global demand for starch-based products [121].

Chitin is primarily extracted from crustacean waste, with industrial production estimated at around 20 kilotons annually [122,123]. The growth in the production of these biopolymers is primarily driven by demand from the food and beverage, agrochemical, and healthcare industries. Furthermore, increasing interest in natural fiber-based biodegradable biopolymers and their emerging applications in fields such as nanotechnology are further pushing market expansion [122].

#### 4.2.1. Cellulose and Nanocellulose

Cellulose is the most abundant natural biopolymer on Earth. It is a primary structural component of plant cell walls and can also be found in some algae, bacteria, and tunicates [124]. Cellulose consists of linear chains of β-(1,4)-linked D-glucosyl units, which assemble into fibrillar structures through extensive hydrogen bonding, resulting in high mechanical strength and chemical stability. Cellulose forms highly ordered structures due to its β-linkages, leading to high crystallinity and limited solubility in water and most organic solvents [125].

**Figure 10:**
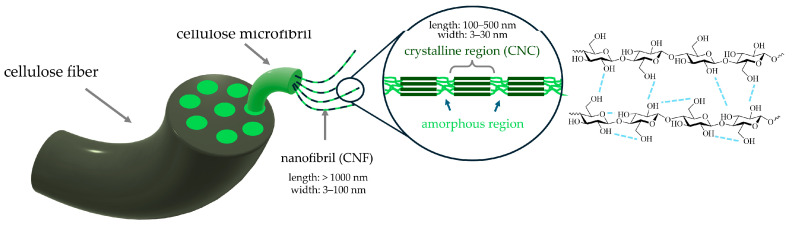
Schematic representation of the cellulose hierarchical structure from plant [126].

Cellulose microfibrils within plant cell walls exhibit a hierarchical structure, consisting of crystalline and amorphous regions. The crystalline regions provide mechanical reinforcement, whereas the amorphous regions introduce flexibility (Figure 10). Depending on the source, cellulose microfibrils can vary in dimensions, crystallinity, and chemical composition, which may include associated hemicelluloses and lignin [126]. Cellulose exhibits amphiphilic properties due to the structure of the glucopyranose ring, where the equatorial positions are hydrophilic, while the axial positions are hydrophobic [127]. The extraction and purification of cellulose involve a series of processes, including mechanical disruption (grinding, milling), chemical or enzymatic treatments (bleaching, delignification), and separation techniques (centrifugation, filtration) to remove non-cellulosic components [128].

Cellulose from plants can be isolated into nanosized particles, commonly referred to as nanocellulose, which has, at least, one nanoscale dimension (<100 nm). There are two main types: cellulose nanocrystals (CNCs) and cellulose nanofibrils (CNFs). CNCs are highly crystalline rod-shaped nanoparticles obtained by acid hydrolysis, which selectively removes amorphous regions while retaining the crystalline domains [129]. They have a high aspect ratio, excellent mechanical properties, and tunable surface chemistry, making them attractive for nanocomposite applications. CNFs are long, flexible fibrils produced by mechanical shearing, enzymatic treatments, or TEMPO-mediated oxidation, preserving both crystalline and amorphous regions [130]. CNFs exhibit a high surface area, good film-forming ability, and shear-thinning behavior, making them suitable for rheology modifiers, coatings, and adhesives.

Alternatively, bacteria such as *Gluconacetobacter xylinus* can generate what is called bacterial nanocellulose (BNC). It is produced through microbial fermentation, either by static or agitated culture, where the former yields structured films while the latter enhances productivity. BNC is a fiber ranging from 20 to 100 nm of diameter which presents a high purity, biocompatibility, and superior mechanical strength, offering excellent tensile properties and flexibility. The cellulose coming from bacteria shows a higher degree of crystallinity, ranging from 80 to 100%, as compared to the wood and plant-based material that range from 40 to 60% [126]. As a sustainable and high-performance material, BNC has broad applications in wound healing, drug delivery, and biocomposites, though its scalability and production costs remain key challenges for industrial adoption [131].

Cellulose and nanocellulose can be functionalized through a series of reactions which utilize the hydroxyl group (Table 4). These hydroxyl groups in cellulose present different reactivity, with C6 being the more reactive. The functionalization can increase the solubility of cellulose, making it more soluble in water or in organic solvent depending on the pendant moiety introduced and the degree of substitution. Amorphous cellulose is more reactive compared to the densely packed crystalline phase [132], for which only groups at the surface are available to be functionalized.

The use of nanocellulose in its various forms, BNC, CNCs, and CNFs, as a colloidal stabilizer in Pickering emulsions and in emulsion polymerization for polymers has been reviewed in 2021 [134]. Since this topic is quite active, several studies have been published since that review. The aim of the following section is to give an overview of the area with a focus on recent developments in this field. Cellulose derivatives are also reviewed as potential biopolymers to be combined with acrylic latexes.

##### Ex Situ Addition

Regarding ex situ strategy, since the previous review, several studies have been published using this methodology for the incorporation of cellulose [135], CMC [136,137,138], HEC [139], modified HEC (HMHEC) [140], CNCs [141,142,143], CNFs [135,144,145,146], and modified CNFs (mCNFs) [146]. The general procedure typically involves preparation of a cellulose solution or dispersion (approximately 0.3–5 wt%) using ultrasound or high-pressure homogenizers to separate the aggregated cellulose. Following this, blending is achieved through the direct physical mixing of both dispersions. Movafagh et al. reported how blending conditions could have an impact on the final latex properties [141]. These authors illustrated that the most significant factors for obtaining enhanced adhesive properties on final carboxylated CNC–acrylic latex blends were mixing speed, sonication, and CNC load.

Direct addition of unmodified and silane-modified CNF powder was reported by Li et al. [146]. The amount of NFCs added was (0.3 wt% base on acrylic latex) into acrylic latex with a solid content of 35 wt% [146]. The mixture was first dispersed by ultrasound for 10 min, and then dispersed by a magnetic stirrer at 600 r/min for 30 min at room temperature. Another example is the study by Ghasemzadeh et al., where CMC was introduced as a thickening agent directly into a diluted acrylic latex (solid content 5 wt%) [138].

##### In Situ Addition

The in situ approach involves polymerizing acrylic latex in the presence of cellulose. When cellulose does not play a key role in colloidal stabilization, surfactants and co-stabilizers are added to the reaction to ensure a stable system.

Grafting cellulose with acrylic monomers is a common strategy, particularly for cellulose derivatives such as CMC [147] and HEC [148]. This can be achieved through direct free radical polymerization using potassium persulfate (KPS) as the initiator. Regarding grafting parameters, a higher initiator concentration decreases GE due to the termination of active hydroxyl radicals formed in the HEC backbone, while temperature exhibits a parabolic trend, initially improving GE and then decreasing [148].

As mentioned, modifying cellulose enhances its solubility in organic solvents, including monomers. Esmaeili et al. dispersed modified CNCs (mCNC) into the monomer mixture before emulsification, followed by emulsion polymerization [149]. However, miniemulsion polymerization offers an interesting alternative, as dispersed or dissolved cellulose remains inside nanosized droplets, enhancing its encapsulation. For miniemulsion polymerization with cellulose acetate butyrate (CAB) [150] and hydrophobically modified CNC [151], increasing cellulose concentration did not affect monomer conversion. However, particle size was influenced due to the increased viscosity of the cellulose–monomer mixture, which reduced the efficiency of droplet breakup during sonication or high-pressure homogenization. In contrast, when ethyl cellulose (EC) was used in miniemulsion polymerization, some phase separation at the o/w interface was reported [152]. Since this was undesirable, the authors resolved it by adding an acrylic crosslinker and an oil-soluble initiator, which promoted grafting and improved overall compatibility between EC and the acrylic polymer.

Alternatively, CNC and CNF dispersions can be charged directly into the reactor, with a pre-emulsion containing the acrylic monomers added dropwise [144,145]. However, Gabriel et al. reported instability during in situ polymerization with carboxylated CNCs due to the high ionic strength of the medium [153]. To obtain stable latexes, the authors added a carboxylated CNC dispersion dropwise into the reactor and partially replaced the ionic surfactant and persulfate salt for a non-ionic surfactant and oil-soluble initiator.

The type and concentration of nanocellulose significantly influence the synthetic process, making each system quite unique and difficult to generalize. Typically, polymerization is carried out in an alkaline medium, with nanocellulose loads range from 0.5 to 1.5 wt% based on monomer weight. Higher concentrations are generally avoided due to challenges associated with high-shear mixing, such as excessive heat generation, during sonication, and the risk of gelation at high nanocellulose concentrations [134]. The presence of nanocellulose in the water or monomer phase prevents the dilution of the final samples observed in the ex situ approach. However, in some cases, it may compromise the stability of the latex.

CNCs and CNFs can stabilize monomer droplets to form a stable emulsion. As previously explained, stabilization using solid particles is referred to as a Pickering emulsion, which does not require the addition of surfactants. Several studies have investigated the use of unmodified CNCs for this purpose. Saelices et al. reported the use of unmodified CNCs as Pickering stabilizers for the emulsion polymerization of styrene and acrylates [154]. Their findings indicated that monomer solubility in the aqueous phase was the key factor influencing the polymerization mechanism. Highly water-insoluble monomers such as lauryl methacrylate (LMA) predominantly formed larger polymer particles (micrometric size), and the process closely resembled a suspension polymerization. In contrast, when sparingly water-soluble monomers such as styrene (ST) and isobornyl acrylate (iBoA) were used, both micro- and nanometric particles were present as a result of the combined suspension and emulsion polymerization. The addition of water-soluble initiators could promote the formation of a larger number of nanometric particles. However, CNCs were not able to stabilize methyl methacrylate (MMA) due to its high solubility in water, which prevented the formation of stable emulsions under similar conditions.

The ability of CNCs to stabilize emulsions without surfactants is advantageous in reducing environmental impact, but is often challenging to achieve and depends on maximizing the interfacial activity of CNCs by functionalization. For example, Zhang et al. carried out Pickering-stabilized miniemulsion polymerization of a 2-ethyl hexyl acrylate copolymer using hexyl-functionalized CNCs to ensure interfacial adsorption [155]. Films cast from the latexes showed huge improvement in mechanical properties, even at low CNC loadings (1 wt%). Errezma et al. carried out Pickering emulsion polymerization using modified aldehyde-functionalized CNCs, obtaining a stable latex at 10 wt% of solid content using a redox system (KPS/Na_2_S_2_O_5_). In addition to generating radicals, the hydrogen sulfite reacted with the aldehyde groups on CNCs to form a bisulfite adduct. In the presence of persulfate, this adduct underwent further reaction to form an active radical, enabling the mCNCs to act as macroinitiators enhancing the stability of the resulting latexes [155]. It should be noted that the presence of persulfate can also generate active radicals at the nanocellulose surface, and therefore, as a consequence, some graft copolymerization can take place [145].

Ionic interactions can also improve the stabilizing effects as reported by Limousin et al. who studied the interaction between anionic CNCs (due to the sulfate groups) with cationic initiators used during the emulsion polymerization [156]. In this approach, the ratio of cationic initiator and CNC was shown to be critical. At lower values, significant aggregation/coagulation of the polymer occurred, whereas at higher values, the CNC did not adsorb to the polymer surface and the final product was effectively a blend of latex and free CNCs. Recent work published by Zhou et al. showed the stabilization effect of CNCs modified by amphiphilic block copolymers [157,158,159]. They showed that the functionalized nanocellulose generally remains at the surface of the particle due to its hydrophilicity, forming a shell surrounding the particles. Alternatively, in other work, it has been shown that hydrophobically modified CNCs can lead to partial or complete internalization of the CNCs within the latex particles [151,160]. Thus, modifying CNCs allows for the synthesis of a distinct range of particle morphologies.

##### Properties and Applications

The influence of cellulosic biopolymers on the physical properties of hybrid materials largely depends on the compatibility between cellulose and the polyacrylate matrix, as well as its proper distribution within the system. Li et al. reported that the inclusion of CNF and mCNF to an acrylic resin led to an increase in tensile strength. However, silane modification of CNF resulted in a greater improvement without significantly compromising elongation at break due to the improved compatibility [146]. Similarly, nanocellulose functionalization has been shown to enhance both tensile strength and elongation at break, especially when it undergoes crosslinking with the acrylic matrix. However, an optimum mCNC concentration exists beyond which both parameters decrease, likely due to aggregation effects [149,157,158,161].

The aspect ratio also plays a significant role in the homogeneity of the distribution. Chiromito et al. reported a comparative study of the addition of cellulose wood pulp and CNFs, demonstrating that the blend containing CNF resulted in a greater improvement in mechanical properties [135]. Regarding the transparency of the hybrid films, acrylic-cellulose systems tend to exhibit good transparency [145,155,156]. The inclusion of hydrophilic cellulose usually increases the water uptake as compared to the pristine acrylic polymer, but it can be minimized with the selection of nanocellulose type and addition method [135,145,146]. However, an enhancement in the hydrophobicity of the surface has been reported as a consequence of the increased roughness [145,161]. Moreover, it has also been shown that by the modification of cellulose, the compatibility can also be enhanced [146,149]. Finally, thermal resistance has generally been observed to increase with the cellulose content [136,145,149,157].

Nanocellulose has been reported to play a key role in improving product performance in a range of applications. For example, the combination of cellulose with acrylic latexes has been demonstrated as a rheology modifier in soil applications [138], antibacterial coatings [139], as a co-solvent for battery separators [136], and as anti-settling agent for road mark paints offering a higher resistance under the operation conditions [137]. Nanocellulose has also been utilized to improve properties in adhesives [141,145,149,151,162,163], paints [143], and paper packaging [144].

In recent years, Zhou et al. have reported a series of studies showing the self-healing properties of fluorinated acrylic latexes, enabled by the incorporation of modified CNC functionalized with coumarin groups. These coumarin functionalized CNCs undergo [2 + 2] cycloaddition under UV irradiation (365 nm wavelength) forming crosslinks that facilitate self-repair [157,158,159]. These latexes were applied to fabrics, imparting hydrophobic and oleophobic properties while also introducing self-healing functionality, which enhances fabric durability by repairing damage [157].

As a final remark in this section, it is worth noting that despite the incorporation of biodegradable cellulose, limited studies about the biodegradation of the hybrid latexes have been conducted. Table 5 provides a summary of the various methods for the preparation of cellulose-based acrylic dispersions and the proposed applications.

#### 4.2.2. Starch

**Figure 11:**
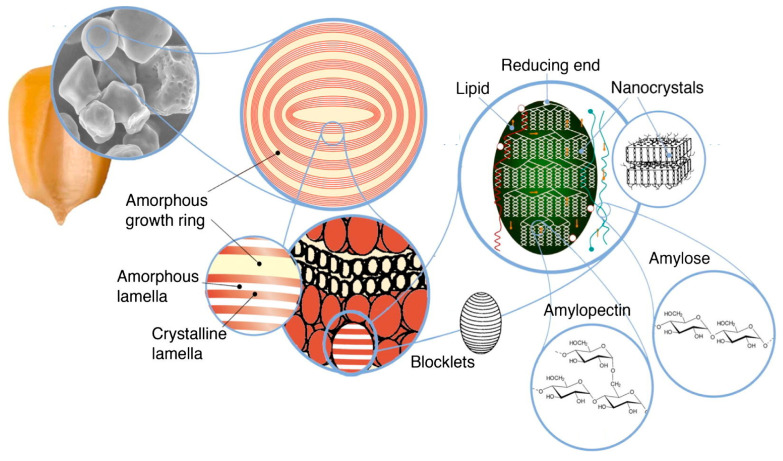
Amylose and amylopectin molecular structure and schematic arrangement of the different structures in the starch granules. Reprinted and adapted with permission from reference [167]. Copyright © 2014 Elsevier.

Starch is produced by plants and can be found in the chloroplasts of green leaves and in the amyloplasts of storage organs such as seed and tubers [164]. This polymer exists in the form of granules, which vary in size from 1 to 100 µm [165], and are primarily composed of amylose and amylopectin (Figure 11). The granules are organized into concentric layers containing both semi-crystalline and amorphous regions, known as granular rings [166].

Amylopectin is responsible for the semicrystalline nature of starch granules. It consists of short chains of α-(1,4)-linked D-glucosyl units, typically ranging from 6 to 35 long glucose units. These chains are connected through α-(1,6)-linkages at their reducing ends, forming highly branched clusters [166]. In contrast, amylose has a molecular weight up to 1 to 3 orders of magnitude lower than amylopectin. It is primarily linear, composed of α-(1,4)-linked D-glucosyl units and is predominantly found in the amorphous region of the starch granules [165]. As is common to many biopolymers, the granule composition and the proportion of amylopectin to amylose changes significantly as a function of the source.

The isolation method and modification of starch is also a function of the source. For example, obtaining starch from cassava and potato tuber is easier than those from cereals because of their low protein and fat content, which means a reduced number of components to be removed [168]. However, the following general steps are required: a first washing of the raw material to remove contaminants, mechanical disruption (e.g., grinding, milling) to release starch granules from the plant cells, separation of starch from other components (which involve sieving, decantation, or centrifugation), purification by repeated washes or with chemical agents, and final drying [168].

The direct use of granules as bio-composites/blends has a limited range of applications due to their large size and inherent hydrophilicity, which promotes their aggregation due to reduced compatibility with the common hydrophobic acrylic polymers [169,170,171,172,173]. For this reason, starch should be modified, either physically or chemically, to be compatible in the hybrid polymer systems to obtain the maximum performance (Table 6). Furthermore, other options where modifications can be obtained by enzymes [174] or directly from the plant through genetic modification [165] exist.

For the use of starch in acrylic emulsion systems, nanoscale starch is especially interesting. There are two types of nano-size starch, which are nanocrystals (SNCs) and nanoparticles (SNPs). The former are crystalline platelets obtained from disrupting the semicrystalline structure of starch granules, while the latter are amorphous.

Both native and modified starch can be used in different fields such as adhesive production, agrochemical, cosmetics, detergent, oil drilling, paper and board, pharmaceuticals, plastics, purification, and textile [164].

Cummings et al. reviewed the use of starch in emulsion polymerization, where they outlined the main synthetic strategies for incorporating starch into latex systems [176]. Building on this work, we will examine the most significant studies in the field, highlighting recent advances and focusing on the properties achieved through the use of starch, as well as its role in the acrylic latexes.

##### Ex Situ Addition

As will be discussed below in more detail, similar to casein, grafting of starch has been the preferred method for its incorporation into acrylic latexes. Consequently, to the best of our knowledge, there are only a few examples in which starch solution was added ex situ as part of an acrylic latex formulation. In these systems, starch has been used as either a thickening agent [139] or a sizing agent [113] or else simply used for comparative purposes against in situ approaches [171].

##### In Situ Addition

Similar to cellulose, for in situ approaches, systems using both grafting (for native starch and its derivatives) and Pickering emulsion polymerization (for starch nanoparticles) have been used.

The primary mechanism used for grafting starch is free radical polymerization. In this process, the generated radical abstracts hydrogen atoms, either from the C-H or O-H bond of the starch backbone, thereby forming a starch macroradical [176]. As a consequence, synthetic factors, such as initiator and monomer concentration, reaction temperature, starch amount and type, affect stability, conversion, and GE.

In grafting systems, the effect of initiator concentration normally presents a parabolic trend, where an initial increase in initiator concentration increases the monomer conversion, GE and PG, until one point where these values decrease or level off due to the excess of radical species that either terminates the growing chains or simply promotes the polymerization of monomers directly [177,178].

An increase in monomer concentration facilitates the diffusion of monomer molecules to the radicals generated on the starch backbone, thereby enhancing conversion, GE, and PG. Furthermore, as monomer concentration increases, ungrafted growing chains can readily react with starch-grafted chains through termination by combination, which also contributes to an increase in conversion, GE, and PG. However, when the monomer concentration increases beyond a certain point, it has been shown that PG remains constant, suggesting that the active sites on the starch molecules are almost fully grafted with vinyl monomers [177]. Higher temperatures increase the conversion and PG until a point that PC starts to decrease [179]. It should be noted that at high temperatures in aqueous media, the starch structure can be affected by swelling with water that can result in a decrease in crystallinity [173].

GE can be improved using SNCs instead of native starch because by reducing the size of the macromolecules, its miscibility with monomers is increased [178]. At low amounts of starch, there is only a limited stabilizing effect. Further increases in starch reduce PC and PG [179] due to the increased viscosity (mass transfer) and the radical scavenging effect of the bio-compound [180]. Through a statistical analysis, Cheng and coworkers determined that the monomer concentration had the most significant influence on the PG, followed by temperature, initiator concentration, and oxidized starch concentration [170].

Pickering emulsion polymerization can also be carried out by using SNCs to stabilize the monomer droplets without the need for any direct grafting of the acrylic monomers [169,171,173]. Hydrolysis reagents can modify nanoparticle surface (e.g., sulfate moieties) increasing their charge, leading a big differences in the obtained particles [169,171].

In emulsion polymerization, viscosity plays a critical role in influencing both mass and heat transfer within the system. When attempting to incorporate a higher amount of starch into hybrid materials, the presence of starch in the water phase significantly increases the viscosity due to its high hydrophilicity and partial solubility. This increase in viscosity can hinder the process, making it more challenging to achieve the desired incorporation of starch. Toward increasing the starch content, Zhang et al. modified SNPs by increasing the crosslink density of the nanoparticles and introducing vinyl moieties with a functionalized sugar-based monomer and a “tie-layer” made of butyl vinyl ether (BVE). This “tie-layer” acted as a bridge between the SNPs and the hydrophobic monomers, obtaining latexes at 42 wt% of solid content and 17 wt% of loaded starch [172]. The addition of either BVE or crosslinked starch SNPs were shown to lead to obtain stable latexes. However, SNP functionalization was necessary to limit formation of grit and to obtain stable, low viscosity latexes. Moreover, SNP functionalization also led to a core–shell structure, where starch remained encapsulated by the acrylic phase, instead of being on the surface as was usually reported. Unfortunately, the low grafting degree of the starch core within the acrylic phase decreased the shear strength compared to pure acrylic film.

Later, the same authors went further in their study evaluating the maximum load of modified SNPs (mSNPs) that can be incorporated into the acrylic emulsion system [180]. Two limiting scenarios were reported: 45 wt% and 25 wt% of mSPNs for 40 wt% and 55 wt% solid content. For each latex solid content, there was a limit on the maximum mSNPs that could be loaded. Latex stability limits were reached due to the lower polymerization rates observed (consumption of radicals by mSNPs) and increased viscosity due to the presence of mSNPs. Despite the improvement on the high bio-content at high solid of mentioned latexes, the high amount of starch resulted in worse adhesive performance of the final hybrid materials. As an alternative to chemical treatment of starch, Cabrera et al. reported the starch modification with hydrophobic zein protein, obtaining combined bioparticles (BPs) by nanoprecipitation, which enhanced the compatibility between starch and acrylic phase allowing a high bio-content (20 wt%) [181].

##### Properties and Applications

Regarding the use of hybrid starch–acrylic emulsions, pressure-sensitive adhesives (PSA) are one of the most frequently targeted applications for the use of starch in combination with acrylic latexes [19,172,173,180,182,183]. Water-based acrylic adhesives are characterized by the use of monomers with low T_g_ because they are soft and tacky at room temperature but also tend to lead to relatively poor shear strength. The addition of starch, which has a higher T_g_, is expected to offer a reinforcing effect (providing some cohesiveness). However, the effectiveness of this reinforcement would be influenced by factors such as the nanofiller’s dispersion efficiency, the interfacial interactions between both phases, and the fractal clustering in the matrix [173].

Zhang et al. focused on obtaining good adhesive properties in acrylic–starch systems by modifying the acrylic phase microstructure by the addition of crosslinker and chain transfer agents [183]. The addition of both agents had a negligible effect on the polymerization kinetics, latex particle size, and viscosity to finally obtain a range of adhesive properties for latexes with 15 wt% of mSNPs at 40 wt% of solid content. Further improvement of the adhesive properties was reported by Garbiel et al. who incorporated dispersed CNCs in situ during the seeded emulsion polymerization involving mSNPs [182]. The presence of CNCs resulted in reinforced PSA that had improved shear strength.

In contrast to the core(mSNPs)–shell(acrylic) structure obtained by Zhang et al., where starch played a limited effect on the final adhesive properties, Ben Ayed and coworkers reported the synthesis of stable latexes an acrylic core/SNC shell morphology [173]. As a result of this morphology, SNPs formed a percolated matrix instead of being dispersed into the acrylic matrix. The final adhesive film had significantly improved peel strength as compared to a comparative acrylic PSA.

In addition to PSAs, acrylic–starch hybrids have been proposed for use in a number of applications. For example, in paper coatings and binders, starch has been incorporated to improve paper printability [184], gloss, picking resistance [170], viscosity control, elasticity [185], blocking resistance, and film formation [181]. Hybrid acrylic–starch systems have also been proposed for use as antibacterial films due to the synergistic effect of cationic starch, tea polyphenols, and 3-(trimethoxysilyl)-propyldimethyloctadecyl ammonium chloride [186]. One potential challenge in many of these systems is that increased water absorption is expected due to the hydrophilicity of starch [184]. However, it has been shown that water uptake/sensitivity could be reduced by grafting hydrophobic monomers such as BA [177] or by trapping the starch phase inside the acrylic phase [181].

In addition to simple starch hybrids, more recently, systems involving more complex biopolymer systems have been explored. For example, Cabrera et al. reported a new system in which hydrophilic starch particles were coated with hydrophobic zein particles to form a biocomposite with enhanced compatibility between the bioparticles and the acrylic phase. Additionally, emulsion polymerization was carried out in the presence of casein as a colloidal stabilizer [187]. The final biopolymer content in the resulting hybrid latexes ranged from 14.4 to 27.5 wt%. Despite the initial starch–zein seed having a size of 50 nm, the final latex particles exhibited a larger size due to the formation of starch–zein aggregates. The study primarily focused on the effects of obtaining these particles through batch or semi-batch emulsion polymerization, with batch emulsion polymerization yielding better results in terms of conversion, particle size distribution, and film homogeneity, as well as a higher grafting concentration. The grafting effect was evident in the solubility and increased thermal resistance. Table 7 provides a summary of the various methods for preparation of starch based acrylic dispersions and the proposed applications.

#### 4.2.3. Chitin and Chitosan

Chitin is the main structural component of insect exoskeletons and the shells of crustaceans and arachnids. It is also present in the cell walls of fungi, molds and yeast, as well as in the cell membranes of bacteria [188]. As occurs with other biopolymers, its industrial isolation changes as a function of the source; in particular, for chitin coming from seafood, it is necessary to demineralize, deproteinize, and remove the pigments [189].

**Figure 12:**
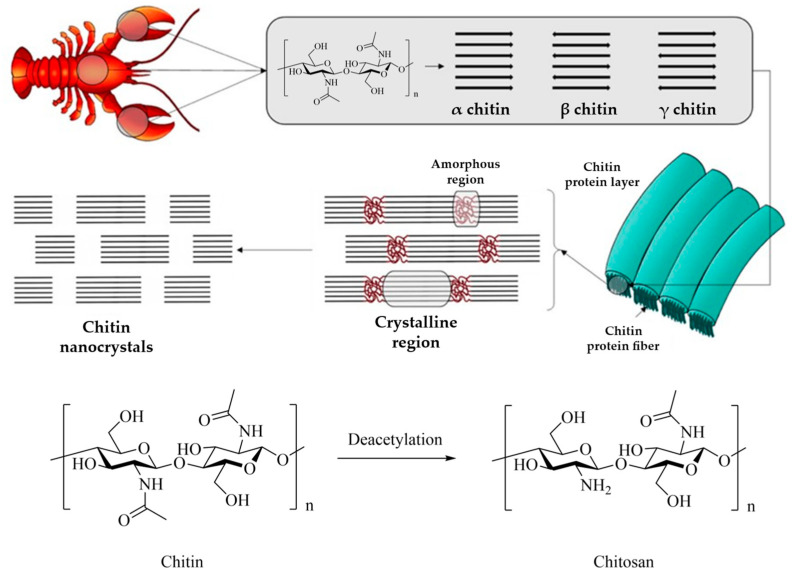
Upper: schematic illustrations for chitin structures and polymorphs, and for the preparations process of chitin nanocrystals and chitosan obtention from chitin deacetylation. Reprinted and adapted from [190]. Lower: deacetylation process for chitosan obtention.

From the chemical point of view, chitin consists of β-glycosidic bonds in the 1,4 position that connects 2-(acetylamino)-2-deoxy-D-glucose units (Figure 12). It is very stable, making it hard to dissolve in water or common solvents. Chitins crystalline structure consists of ordered crystalline microfibrils, with three polymorphs, α, β, and γ which differ in the way the chains are packed, the degree of hydration, the size of the elementary cells, and the amount and degree of polymerization [188]. The α form is the most widespread as it comes mainly from shells of crustaceans, skeletons of insects, which is the main industrial source.

Chitosan is derived from chitin that is produced by deacetylation (Figure 12). The degree of deacetylation, defined as the number of -NH_2_ groups formed compared to the initial number of -NH-CO-CH_3_ groups, has a high impact on the solubility in aqueous acidic solutions, the degree of swelling in water, biodegradability, and bioactivity. This deacetylation process decreases the molecular weight and crystallinity of chitin from being in excess of 1 million to somewhere in the range of 100,000 to 1,200,000 with low crystallinity. Regarding the solubility, chitosan is soluble at a pH lower than 6.2. This biopolymer can be easily modified by means of different procedures (Table 8) due to the presence of amino and hydroxyl groups in its structure.

Chitosan has been used in different industries such as cosmetic, packaging, medicine (tissue engineering, antibacterial activity hemostatic design), agriculture, water and wastewater treatments, textile, and paper.

##### Ex Situ Addition

Regarding ex situ approaches to acrylic hybrids, these have primarily been employed for the dispersion of chitosan complexes with metals and metal oxides [192,193,194,195], as well as modified chitosan [196,197]. These complexes or modified chitosan are pre-formed and introduced directly into the acrylic latex. The primary role of chitosan in this context is to enhance the dispersion of the metallic ions and nanoparticles in water and within the final polymer after film casting.

Torabi et al. reported the preparation of ex situ blends containing high chitosan loads (up to 40 wt%) in an acrylic emulsion stabilized with a cationic surfactant at a low solid content (10 wt%) [198]. Furthermore, the presence of epoxy groups from glycidyl methacrylate allowed a reaction with the amine groups of chitosan during the drying process, leading to crosslinking of both polymers [198]. Similarly, Promlok et al. reported the direct addition of a chitosan solution into a hybrid latex made of acrylic-modified natural rubber latex [199].

Although not involving an emulsion, Zhong described the incorporation of chitin nanocrystals (ChNCs) into a water-soluble acrylic resin (Joncryl 678). In this work, a dispersion of ChCNs in alkaline solution was used to dissolve the acrylic resin [200], enabling the preparation of a formulation containing up to 5 wt% ChNCs with a solid content of 26–27 wt%.

##### In Situ Addition

In terms of in situ polymerization, several factors affect the latex properties and conversion. As reported by Yang et al., the selection of the initiator is a key factor to obtain a stable emulsion [201] as charged chitin fibrils (ChNFs) interact with charged initiators. When an initiator with the same charge was used, stable latexes were obtained due to the repulsive interactions between colloidal acrylic particles and fibrils. On the other hand, when an initiator with opposite charge was used, despite achieving full conversion, the product coagulated. In the same study, CNCs were also tested under the same conditions as ChNFs. It was observed that latexes incorporating ChNFs exhibited a narrower size distribution compared to those with CNCs [201]. It was concluded that nucleation occurs in the aqueous phase, allowing the undissolved monomer to diffuse toward the growing particles. The small particle size and narrow size distribution obtained evidenced that emulsion polymerization, rather than suspension polymerization, dominated the latex formation [201].

Wada and coworkers reported a feasible strategy to introduce chitosan at concentrations of up to 5 wt% through pre-mixing it with acrylic acid to obtain a chitosan–acrylate salt [202,203,204]. They conducted a semi-batch emulsion polymerization comparing two feed strategies: a pure monomer mixture and a pre-emulsion. The direct addition of the monomer mixture resulted in an unstable emulsion with high viscosity and partial precipitation. In contrast, the pre-emulsion method produced a stable emulsion with a solid content of 34 wt% [203]. The type of acrylic acid monomer (AA, MA, or ItA) used to obtain the chitosan salt had a strong influence in the latex final pH and viscosity and in the incorporation of the chitosan which led to different film homogeneity [204]. However, the incorporation of the chitosan–acrylate salt negatively impacted the mechanical properties. As Wada et al. reported, this salt can reduce the molecular weight of the resulting acrylic polymer, leading to a deterioration in tensile strength and elongation at break compared to the pure acrylic formulation [203]. Latexes containing quaternized chitosan (q-chitosan) exhibited more homogeneous films and significantly lower viscosity (100 times lower) to those with chitosan, which was attributed to the enhanced solubility of q-chitosan in aqueous acrylic systems [202]. As expected for a biopolymer, chitosan’s molecular weight significantly influences the final latex properties; an increase in M_w_ raises the final viscosity as well as the amount of water absorption and the heterogeneous distribution within the acrylic matrix [204]. Addressing the water absorption problem, it has been reported that a reduction in water sensitivity can be achieved by incorporating diacetone acrylamide (DAAM), which can crosslink with both the carbonyl groups in the acrylic phase and the amino groups in chitosan [202].

Pickering emulsions stabilized by chitosan were reviewed by Meng et al. [205]. Particles arising from the self-assembly of chitosan can be formed by controlling the pH of chitosan solution. These particles serve as effective stabilizers for Pickering emulsions due to their amphiphilic properties, but no reported works have been found for its use in emulsion polymerization.

As previously explained, Li and coworkers studied the direct grafting of acrylic monomers onto various biopolymers that contain -NH_2_ moieties using tBHP as the initiator [82]. While the polymerization resulted in high monomer conversion, lower GE was observed for chitosan. This was attributed to the free-radical decomposition of tBHP, which was promoted by the presence of acetic acid used to dissolve chitosan. This effect enhanced the homopolymerization of the acrylic monomer, reducing the efficiency of the grafting process (52%). Pradhan et al. reported the grafting of chitosan with PMMA by emulsion polymerization using as initiator ammonium persulfate with a complex of CuSO_4_ and glycine (1:1) and a sorbitol surfactant, obtaining a 89.6% of grafting [206].

##### Properties and Applications

The incorporation of chitin and chitosan into acrylic latex is not exclusively intended to increase the bio-content of the final hybrid material. Due to their unique structure, a significant number of studies have explored additional functionalities of these polysaccharide-containing latexes. These include their ability to absorb gases, particularly formaldehyde [199,202,203,204], to prepare antibacterial and antifouling coatings [193,198,202], and non-halogenated fire-proof coatings [196,197].

Chitosan has the ability to absorb formaldehyde as the result of the reaction between amino groups of chitosan and carbonyls of formaldehyde. When a Schiff base is formed, the adsorbed formaldehyde is not released even when the temperature is raised [207]. This property can be achieved by direct ex situ wet blending of chitosan [199,203], but improved activity absorbing hydrogen sulfide and ammonia was reported when in situ hybrid chitosan–acrylic latex was obtained compared to direct film-dispersed chitosan [203]. The adsorption of formaldehyde is directly proportional to the available amino groups and therefore a decreased adsorption activity was reported when derivatives such as q-chitosan are used [202].

Amino groups in chitosan interact with negatively charged residues on the macromolecules of bacterial cell surfaces, thereby inhibiting their growth. It is important to note that this antimicrobial behavior is only observed in acidic media. Different antibacterial activity has been reported for hybrid coating q-chitosan–acrylic compared to chitosan–acrylic. Both coatings effectively eliminated *Escherichia coli*, but the q-chitosan–acrylic coating demonstrated greater effectiveness against *Staphylococcus* [202]. Jin-Lan et al. prepared an antibacterial carboxymethyl chitosan complex (Ag-carboxymethyl chitosan–thiabendazole) which was blended with an acrylic water-based paint [193]. The addition of 0.1% of this complex did not change the color, luster, viscosity, odor, or pH value, but presented persistent broad-spectrum antimicrobial activity. Limited antibacterial activity was found for acrylic latexes obtained by Pickering emulsion polymerization using ChNFs as colloidal stabilizer, which was enhanced by the incorporation of miconazole nitrate [201].

Chitosan has been reported to offer some benefits as a fire retardant due to the high ash generation that acts as insulator for the mass transfer [198]. Carboxymethyl chitosan modified with a silane coupling agent (KH550) was shown to enhance its ability to form a stable complex with boron nitride, resulting in a composite that showed improved compatibility and dispersion within the silicone acrylic phase [197]. This complex could be incorporated into silicone acrylic emulsions to enhance the fireproof performance of the coating. Further work has been reported using carboxymethyl chitosan to improve the compatibility between a silicone acrylate emulsion matrix with melamine polyphosphate and sodium lignosulfonate (LS) [196]. This composite showed improved flame retardancy and smoke suppression. It was reported that the crosslinking of LS and carboxymethyl chitosan–melamine polyphosphate complex (CMPP) provided a synergistic enhancement in flame retardancy. Additionally, the adhesion of LS and the filling effect of CMPP contributed to the formation of a dense and continuous carbon layer during combustion [196].

Antifouling properties have also been reported for polycationic chitosan [208]. Abiraman et al. reported a series of articles addressed to the antifouling properties offered by the incorporation of chitosan complexes with different metals: zinc oxide [192], copper oxide [195], and copper nanoparticles [194]. These complexes could be easily dispersed into acrylic paints without disrupting the emulsion. Table 9 provides a summary of the various methods for preparation of chitosan- and chitin-based acrylic dispersions and the proposed applications.

## 5. Conclusions

This review has presented an overview of the research that has been conducted on the combination of biopolymers including casein, soy protein, collagen, gelatin, cellulose, starch, chitin, and chitosan with waterborne acrylic latexes. It has been shown throughout that the strategy used to integrate these components is crucial in obtaining the desired properties.

Ex situ blending is a simple and straightforward approach, where biopolymers are typically dispersed in water and subsequently mixed with waterborne acrylic latex systems. However, the resulting hybrid latexes tend to be more diluted, which may compromise their end-use applications. Conversely, in situ addition involves conducting the polymerization of waterborne acrylic latexes in the presence of the biopolymer. This approach presents greater challenges, as colloidal stability can be compromised. The use of radical initiators during the synthesis facilitates the grafting of acrylic monomers onto the biopolymer backbone, as biopolymers tend to have reactive functional groups, such as carboxylic acids, amines, and alcohols, that are susceptible to hydrogen atom abstraction from radical initiators. Formation of radicals in the biopolymer structure can lead to grafting by reaction with double bonds of acrylic monomers, but high biopolymer loads can also promote a radical scavenger effect, which leads to inhibition/retardation of the polymerization. The grafting effect can be enhanced by optimizing the initiator system, for example, by using tBHP in casein to form a redox pair with amine moieties, or by functionalizing the biopolymer to introduce reactive groups. Compared to the ex situ approach, in situ hybrid latexes generally demonstrate enhanced compatibility between the biopolymer and synthetic polyacrylate, which generally results in enhanced physical properties.

Due to the inherent hydrophilicity of biopolymers, in in situ methods, core–shell structures are commonly formed, with biopolymers surrounding the polyacrylic particles. In some cases, the amphiphilic nature of biopolymers leads to surfactant-like behavior which permits emulsifier-free emulsion polymerization to be conducted. For polysaccharides, the high surface activity of nanocrystals and nanofibrils often allows for Pickering stabilization, which makes Pickering emulsion polymerization routes to biopolymer/acrylic hybrids possible.

Generally, the film formation process is not significantly affected by the addition of biopolymers, although it can promote film formation in some cases due to hydroplasticization effects. The distribution of biopolymers within the polyacrylic matrix depends on the compatibility between both phases. Enhanced compatibility will promote the even-dispersion of the biocompounds, which can be achieved by increasing the percentage of grafting and increasing the hydrophobicity of the biopolymer through chemical modifications.

A broad range of properties has been reported for these hybrid materials, including improved colloidal stability, barrier properties, antifouling, antibacterial activity, formaldehyde absorption, and flame retardancy. Moreover, the presence of biopolymers enhances the biodegradability of the final hybrid film. As a result, several end applications including adhesives for wood, PSA, paints, textile, paper, and construction have been proposed for these new hybrid materials. Recent studies have explored the combination of multiple biopolymers, each serving a distinct role, such as acting as a colloidal stabilizer or enhancing specific properties. These approaches illustrate how biopolymers can function as a versatile and eco-friendly toolbox for synthesizing and modifying waterborne acrylic latexes.

Regarding the availability of biopolymers and their immediate potential for large-scale implementation in acrylic dispersions, there are a number of feasible options. Casein and collagen are produced at commercial scale and their use as colloidal stabilizers to substitute petroleum-based surfactants could in principle be adopted by industry. However, their limited implementation may be attributed to the need to functionalize their structure to increase the compatibility with acrylic monomers. Although only limited studies of soy/acrylic hybrid latexes have been reported to date, soy protein is produced in high volumes and therefore presents a good option for large-scale applications. Cellulose derivatives such as CMC, HEC, and starch are also commercially available on a scale that is sufficient to meet the demands of the waterborne acrylic dispersion industry. In contrast, the industrial production of polysaccharides nanomaterials is not yet fully established. However, technological developments are expected to improve their production volumes, enabling cost reductions and more standardized materials.

The production of waterborne acrylic latexes is continuously evolving to balance the demand for high-performance materials with increasing environmental and sustainability concerns. Emulsion polymerization provides a more environmentally friendly production route, while the integration of biopolymers and renewable monomers has opened new avenues for developing more sustainable high bio-content (meth)acrylic-based materials. Further advancements in sustainability have been achieved by incorporating bio-based acrylates, with reported biomaterial content reaching up to 75 wt%. As research progresses, future innovations are expected to focus on enhancing the biocompatibility, biodegradability, and functional versatility of polyacrylic systems to meet the growing demands of an eco-conscious market.

## Figures and Tables

**Table 1:** Comparison of cited works for the combination of casein with waterborne acrylic latex.

Approach	Biopolymer		Acrylic Composition	Synthesis	Initiator	Role of Biopolymer	Applications	Ref.
	Type	wt% Based on Monomer						
Ex situ	Casein Gelatin	14.5–40 *	MMA/BA/AA/AAEM	Physical mixing	-	Crosslink agent	Coatings	[74]
In situ	Casein	3–16 × 10^−4^ * mol casein/mol monomer	BA MA EMA	Batch EF-EP	KPS KPS/Ascorbic acid	Colloidal stabilizer Additive	Leather treatment	[77,78,79,80]
In situ	Casein Gelatin Chitosan	25 *	MMA	Batch EF-EP	tBHP	Colloidal stabilizer	Nanoparticle design	[82]
In situ	Casein	17–100 *	MMA	Batch EF-EP	tBHP, KPS, AAPH, AIBN, BPO, TBP, CHP	Colloidal stabilizer	Nanoparticle design	[81]
In situ	Casein	3–50	MMA	Batch EF-EP	tBHP	Colloidal stabilizer	Coatings	[83]
In situ	Casein	3–50	BA/MMA	Batch EF-EP	tBHP	Colloidal stabilizer Additive Improve biodegradability	Binder	[94]
In situ	Casein	20 *	MMA/BMA	Batch EF-EP	tBHP	Colloidal stabilizer Phase compatibilizer	Dipped products	[95]
In situ	Casein	3–50	BA	Batch EF-EP	tBHP	Colloidal stabilizer Additive Improve biodegradability	Food packaging	[84]
In situ	Casein-CPL	67 *	BA/MMA	EF-EP	APS	Colloidal stabilizer	Coatings	[85]
In situ	Casein-CPL	70 *	BA	Batch EF-EP	APS KPS APS/NaHSO_3_ KPS/NaHSO_3_	Colloidal stabilizer Improve biodegradability	Binder Leather treatment	[86]
In situ	Casein-CPL	67 *	BA/MMA/VAc	Batch EF-EP	APS	Colloidal stabilizer	Ink binder	[88]
In situ	Casein-CPL	140 *	BA/MMA/Vi-PDMS	Semi-batch EF-EP	APS	Colloidal stabilizer	Adhesive	[87]
							Textile	
							Food packaging	
In situ	Methacrylated casein	25	BA/MMA	Batch EF-EP	tBHP	Colloidal stabilizer Additive	Coatings	[90]
In situ	Methacrylated casein	25–50	BA/MMA	Batch EF-EP	tBHP	Colloidal stabilizer Additive	Paints	[93]
In situ	Casein Methacrylated casein	25	IBOMA/2OA/MMA/BA	Batch EF-EP	tBHP	Colloidal stabilizer	Coatings	[91]
In situ	Methacrylated casein	25	BA/MMA	EF-EP	KPS	Colloidal stabilizer Additive	Coatings	[92]
In situ	Casein/zein	20–60	BA/MMA	Batch EF-EP Semi-batch EF-EP	KPS	Colloidal stabilizer Additive	Coatings	[96]

* Calculated value based on the reported data.

**Table 2:** Comparison of cited works for the combination of soy protein and waterborne acrylic latex.

Approach	Biopolymer		Acrylic Composition	Synthesis	Initiator	Role of Biopolymer	Applications	Ref.
	Type	wt% Based on Monomer						
Ex situ	Soy meal	600–3000 *	Commercial acrylic emulsion	Physical mixing	-	Structural component	Adhesive (wood)	[49]
Ex situ	Modified SPI	0–900 *	BA/MMA	Physical mixing	-	Structural component	Adhesive (plywood)	[101]
In situ	Soy protein	1–5	PMMA	Batch EF-EP	KPS	Oxygen barrier Flame retardant Improve biodegradability	Packaging	[97]
In situ	Modified soy protein	1.8 *	BA/VAc/MMA/AA	EP	APS	Reinforcement	Adhesive (wood)	[105]
In situ	Soy protein Modified soy protein	1.3–6.3 *	BA/MMA	Batch MEP	APS	Additive Protective colloid	Adhesive (plywood)	[102]
In situ	SPI	4–5.5 *	BA/MMA/AAEM	Semi-batch EP	APS	Additive UV curing agent	UV curing coatings (wood)	[103]
In situ	SPI	2.6–15.4 *	BA/MMA/AAEM	Semi-batch EP	APS	Reinforcement	Wood coatings	[104]

* Calculated value based on the reported data.

**Table 3:** Comparison of cited works for the combination of collagen with waterborne acrylic latex.

Approach	Biopolymer		Acrylic Composition	Synthesis	Initiator	Role of Biopolymer	Applications	Ref.
	Type	wt% Based on Monomer						
Ex situ	Casein Gelatin	14.5–40 *	MMA/BA/AA/AAEM	Physical mixing	-	Crosslink agent	Coatings	[74]
In situ	Gelatin	25–350 *	AA, AAm, MMA and VAc	EF-EP	KPS	Colloidal stabilizer	Not specified	[111]
In situ	Gelatin	33.3–133.3 *	MA/VAc	Batch EF-EP	APS	Colloidal stabilizer Sizing agent	Paper	[112]
In situ	Gelatin	166.7 *	MAA	Batch EF-EP	UV photoinitiator	Colloidal stabilizer Sizing agent Improve biodegradability	Paper	[116]
In situ	Collagen	150 *	EA/ST	Batch EF-EP	APS	Colloidal stabilizer Sizing agent	Paper	[113]
In situ	HC	15–50	BA/AA	Batch EF-EP	H_2_O_2_	Colloidal stabilizer Additive	Adhesives	[114]
In situ	Methacrylated HC	25	BA/AA	Batch EF-EP	KPS	Colloidal Stabilizer Additive	Adhesives	[115]

* Calculated value based on the reported data.

**Table 4:** Main functionalization of cellulose and nanocellulose [126,133].

Linkage	Reaction	Derivatives
Ethers	Reaction with chloroalkanes (e.g., methyl chloride, monochloroacetic acid) previously treated in alkali media	methyl cellulose (MC) carboxymethyl cellulose (CMC)
	Reaction with ethylene and propylene oxide previously treated in alkali media	hydroxyethyl cellulose (HEC)
	Reaction with epoxides	
Esters	Acetylation by acetic anhydride in acetic acid media and catalyzed by sulfuric acid or perchloric acid	Cellulose acetate (CA)
	Cellulose treated with nitric acid	Nitrocellulose
	Cellulose treated with reagents such as sulfuric acid, chlorosulfonic acid, or sulfur trioxide	Cellulose sulfate
	Reaction with acid anhydrides	
Oxidized	TEMPO-mediated hypochlorite oxidation to introduce carboxylic groups	Oxidized cellulose
Silylated	Reaction with chlorosilanes	Silylated cellulose
Urethane	Reaction with isocyanates	Urethane cellulose
Grafting	Formation of polymer chains on cellulose backbone through free radical, reversible addition fragmentation chain transfer or atom-transfer radical polymerization route	

**Table 5:** Comparison of cited works for the combination of cellulose with waterborne acrylic latex.

Approach	Biopolymer		Acrylic Composition	Synthesis	Initiator	Role of Biopolymer	Applications	Ref.
	Type	wt% Based on Monomer						
Ex situ	Cellulose fiber CNF	33.3–300 *	MMA	Physical mixing	-	Structural component	Fiberboards	[135]
Ex situ	CMC	Added as 0.5 wt% solution	BA/AA/ST	Physical mixing	-	Co-solvent Thickening agent	Adhesive/binder for battery separators	[136]
Ex situ	CMC	0.5 wt% in formula	Commercial acrylic emulsion	Physical mixing	-	Anti-sediment agent	Paints (road marks)	[137]
Ex situ	CMC	1–5 wt% in formula	BA/MMA	Physical mixing	-	Thickening agent	Soil treatment	[138]
Ex situ	HEC	0.8–1.5 wt% in formula	Commercial acrylic latex	Physical mixing	-	Thickening agent	Textile	[139]
Ex Situ	Modified HEC	0.2–0.8 wt% in formula	Commercial acrylic latex	Physical mixing	-	Thickening agent	Paints	[140]
Ex situ	CNC	0.3–1	Acrylic latex	Physical mixing	-	Reinforcement	Adhesives	[141]
Ex situ	CNC	2.5–20	BA/MMA	Physical mixing	-	Reinforcement	Coatings	[142]
Ex situ	CNC	0.5 wt% in formula	Commercial acrylic latex	Physical mixing	-	Crosslinking and anticorrosion agent	Paint	[143]
Ex situ In situ	CNF	0.5–1 0.25–1	2EHA/AA/ST	Physical mixing MEP	APS	Reinforcement	Paper packaging	[144]
Ex situ In situ	CNF	0.5–1 0.5–1	BA/MMA/AA	Physical mixing MEP	KPS	Reinforcement	Wood adhesives Fabric coatings	[145]
Ex situ	Modified CNF	0.3 wt% based on latex	Commercial acrylic latex	Physical mixing	-	Reinforcement	Coatings	[146]
In situ	CMC	7–9 *	BA/MMA/DFMA/VAc	Semi-batch EP	KPS	Reinforcement	Paper	[147]
In situ	HEC	-	AA/MBA	Batch EP	KPS	Reinforcement	Adhesive	[148]
In situ	CAB	20–100 *	BA/MMA/AA	Batch MEP	KPS	Reinforcement Colloidal stabilizer	Nanoparticles design	[150]
In situ	EC	5–20	BA/MMA	Batch MEP	APS LPO	Reinforcement	Coatings	[152]
In situ	CNC	1	BA/MMA	Semi-batch EP	KPS/AIBN	Reinforcement	Adhesive	[153]
In situ	CNC	10–30	MMA/BA	Batch EF-EP	AIBA	Colloidal stabilizer Reinforcement	Coatings	[156]
In situ	CNC	0.2–2.3	iBoA, MMA, BMA, LMA, ST	PEP	AIBN ACPA	Colloidal stabilizer	Not specified	[154]
In situ	CNC	1	BA/MMA	Semi-batch EP	KPS	Reinforcement	Adhesive	[162]
In situ	CNC	0–1.5	2EHA/MMA/ST	Semi-batch EP	NaPS	Reinforcement	Adhesive	[163]
In situ	Modified CNC	0.5–1.5	BA/MMA/HEMA	Batch EP	KPS	Reinforcement	Adhesives	[149]
In situ	Modified CNC	0.5–1.5	BA/AA/VAc	Batch MEP	KPS	Additive	Adhesive	[151]
In situ	Modified CNC	0.5–10	BMA	PEP	KPS/Na_2_S_2_O_5_	Colloidal stabilizer Reinforcement	Binder Adhesive	[155]
In situ	Modified CNC	0.4–1 *	BA/MMA/HFBA/CMA	PEP	KPS	Colloidal stabilizer photo-responsive agent Reinforcement	Textile	[157]
In situ	Modified CNC	0.6–1.4 *	BA/SA/HFBA/MMA/UPyMA/CMA	PEP	APS	Colloidal stabilizer Reinforcement Self-healing agent	Textile Self-healing coatings	[158,159]
In situ	CNC Modified CNC	0.5–1	BA/MMA	Batch MEP	KPS	Reinforcement	Coatings	[160]
In situ	Modified CNC	17 *	BA/MMA/HFBA	Semi-batch EF-EP	APS	Colloidal stabilizer Reinforcement	Textile	[161]

* Calculated value based on the reported data.

**Table 6:** Chemical and physical modifications of starch for non-food industrial purposes [174].

Type	Process	Modification
Chemical	Hydrolysis	The hydrolysis of glycosidic bonds occurs preferentially in amorphous regions, which are more susceptible than crystalline regions. This process is commonly carried out using mineral acids.
	Oxidation	Oxidation of hydroxyl groups to form carbonyl and carboxyl groups using oxidizing agents.
	Crosslinking	Formation of covalent bonds between different starch chains by targeting hydroxyl groups.
	Esterification	Esterification of hydroxyl groups through treatment with organic and inorganic acids.
	Etherification	The formation of ether linkages from hydroxyl groups. Depending on the introduced functional group, cationic, anionic, amphoteric, or non-ionic starches can be obtained.
	Grafting	Formation of polymer chain covalent bonds attached to starch chains. There are several strategies that have previously been reviewed [175].
Physical	Thermal	Involves disrupting the starch intermolecular bonds in water and heat to produce pre-gelatinized and granular cold-water-swelling starches.
	Non-thermal	Process such as milling, ultrasounds, microwave, pulsed electric field, freezing and thawing, and high pressure, which rearrange starch granules, altering their functional properties.

**Table 7:** Comparison of cited works for the combination of starch with waterborne acrylic latex.

Approach	Biopolymer		Acrylic Composition	Synthesis	Initiator	Role of Biopolymer	Applications	Ref.
	Type	wt% Based on Monomer						
In situ	Acetylated cationic starch	59–111 *	BA/ST	Semi-batch EF-EP	FeSO_4_/H_2_O_2_	Colloidal stabilizer Structural component	Paper	[177]
In situ	Starch	20–50	BA/MMA/DAAM	Semi-batch EF-EP	KPS	Colloidal stabilizer Absorbent agent	Indoor coatings (humidity control) Paints	[179]
In situ	Starch	2.8 *	AA/VAc	Semi-batch EP	APS	Filler (Increased bio-content) Crosslinkable functionality	Adhesive (wood)	[19]
In situ	Starch	7–11 wt% based on formula	MMA/ST/cationic acrylate MMA/ST/BA MMA/ST/EA MMA/ST/2EHA	Semi-batch EF-EP	APS	Colloidal stabilizer	Paper	[184]
In situ	Starch	50 *	BA/AA/ST	Semi-batch EF-EP	AAPH	Colloidal stabilizer	Paints	[185]
In situ	Starch	67 *	BA/ST	Semi-batch EF-EP	FeSO_4_/H_2_O_2_	Colloidal stabilizer Antibacterial	Antibacterial coatings	[186]
In situ	SNP	2–10*	BMA	PEP	KPS	Colloidal stabilizer Reinforcement	Coatings Adhesives	[169]
In situ	Modified SNP	15–60 wt% total mass of solids	BA/MMA/AA	Semi-batch EP	KPS	Filler (Increased bio-content)	Adhesive	[180]
In situ	Modified SNP CNC	6–12 0.5–1	nOA/AA/ST	Semi-batch EP	KPS	Filler (Increased bio-content)	Adhesives	[182]
In situ	Modified SNP	15	BA/MMA/AA	Semi-batch EP	KPS	Filler (Increased bio-content)	Adhesive	[183]
Ex situ In situ	SNC	2–12 *	BMA	PEP	KPS	Colloidal stabilizer Reinforcement	Coatings Adhesives	[171]
In situ	SNC	4–10	BMA BMA/EHA	PEP	Citric acid/H_2_O_2_	Colloidal stabilizer Reinforcement	Adhesives	[173]
In situ	Starch/zein	25 *	BA/MMA	Semi-batch EP	KPS	Reinforcement	Binder	[181]
In situ	Casein Starch/zein	5 11–25 *	BA/MMA	Batch EF-EP Semi-batch EF-EP	KPS	Colloidal stabilizer Reinforcement	Coatings Adhesives	[187]

* Calculated value based on the reported data.

**Table 8:** Main chitosan derivative [188,189,191].

Process	Modification
Deacetylation	Alkaline hydrolysis, generally, using sodium hydroxide at high temperatures [191].
Carboxymethyl chitosan	O- and N-carboxymethylation of chitosan by sodium monochloracetate in presence of sodium hydroxide [189].
Aminoethyl chitosan	Reaction with ethylene oxide.
Quaternized chitosan	Quaternary ammonium groups (-NR_3_^+^) added to the chitosan backbone, generally, by quaternary ammonium salts.
Thiolated chitosan	Thiol groups (-SH) are incorporated into the chitosan backbone, typically by thiolating agents.

**Table 9:** Comparison of cited works for the combination of chitin and chitosan with waterborne acrylic latex.

Approach	Biopolymer		Acrylic Composition	Synthesis	Initiator	Role of Biopolymer	Applications	Ref.
	Type	wt% Based on Monomer						
Ex situ	Chitosan	0.06 * wt% in formula	Commercial acrylic paint	Physical mixing	-	Capping agent	Antifouling coatings	[192,194,195]
Ex situ	Chitosan	11.1–66.7 *	BA/MMA/GMA	Physical mixing	-	Antibacterial agent	Antibacterial films	[198]
Ex situ	Chitosan	4.1 wt% based on total polymer	Natural rubber/acrylic hybrid latex	Physical mixing	-	Formaldehyde absorption	Paints	[199]
Ex situ	Carboxymethyl chitosan	0.1 wt% in formula	Acrylic emulsion	Physical mixing	-	Chelating agent	Antibacterial and antifouling paints	[193]
Ex situ	Carboxymethyl chitosan	0.5 * wt% in formula	Silicone acrylic emulsion	Physical mixing	-	Flame retardant	Coatings (wood) Paints (wood)	[196,197]
In situ	Quaterrnized chitosan	5 wt% in solids	MMA/2EHA/AA DAAM	Semi-batch EP	AAPH	Formaldehyde absorption Antibacterial Crosslinkable functionality	Coatings	[202]
In situ	Chitosan	3–10 wt% in solids	MMA/2EHA/AA MMA/2EHA/ItA MMA/2EHA/MA	Semi-batch EP	AAPH	Formaldehyde absorption	Interior finishing coatings	[203,204]
In situ	Chitosan	-	PMMA	Batch EP	APS/CuSO_4_:Glycine (1:1)	Reinforcement Biocompatible matrix	Bioadhesive	[206]
In situ	ChNF CNF	1.8 *	2EHA/MMA	PEP	VA-044 KPS	Colloidal Stabilizer Reinforcement Antibacterial	Adhesive	[201]

* Calculated value based on the reported data.

## Abbreviations

The following abbreviations are used in this manuscript:

2EHA	2-ethylhexyl acrylate
2OA	2-octyl acrylate
AA	acrylic acid
AAEM	acetoacetoxyethyl methacrylate
AAm	acrylamide
AAPH	2,2′-azobis(2-amidinopropane) dihydrochloride
ACPA	4,4′-azobis(4-cyanovaleric acid)
AFM	atomic force microscopy
AIBA	2,2′-azobis(2-methylpropionamidine) dihydrochloride
AIBN	2,2′-azobis(2-methylpropionitrile)
APS	ammonium persulfate
BA	butyl acrylate
BMA	butyl methacrylate
BPO	benzoyl peroxide
BPs	bioparticles
CAB	cellulose acetate butyrate
ChNC	chitin nanocrystal
ChNF	chitin nanofibril
CHP	cumene hydroperoxide
CMA	7-(2-methacryloyloxyethoxy)-4-methylcoumarin
CMC	carboxy methyl cellulose
CMPP	carboxymethyl chitosan–melamine polyphosphate complex
CNC	cellulose nanocrystal
CNF	cellulose nanofibril
CPL	caprolactam
DAAM	diacetone acrylamide
DFMA	dodecafluoroheptyl methacrylate
EA	ethyl acrylate
EF-EP	Emulsifier-free emulsion polymerization
EP	emulsion polymerization
GMA	glycidyl methacrylate
HBP	4-hydroxybenzophenone
HC	hydrolyzed collagen
HEMA	2-hydroxyehtyl methacrylate
HFBA	hexafluorobutyl acrylate
iBoA	isobornyl acrylate
IBOMA	isobornyl methacrylate
ItA	itaconic acid
KPS	potassium persulfate
LMA	lauryl methacrylate
LPO	dilauroyl peroxide
LS	sodium lignosulfonate
MAA	methacrylic acid
MCC	micellar casein concentrate
MEP	miniemulsion polymerization
MFFT	minimum film formation temperature
MHC	Methacrylated hydrolyzed collagen
MMA	methyl methacrylate
NaPS	sodium persulfate
nOA	*n*-octyl acrylate
o/w	oil-in-water
PSA	pressure sensitive adhesive
SNC	starch nanocrystal
SNP	starch nanoparticle
SPI	soy protein isolate
ST	styrene
TA	tannic acid
tBHP	*tert*-butyl hydroperoxide
TBP	*tert*-butyl peroxide
UPyMA	2-Ureido-4[1H]-pyrimidinone methyl methacrylate
VA-044	2,2′-azobis [2-(2-imidazolin-2-yl)propane] dihydrochloride
VAc	vinyl acetate
Vi-PDMS	polydimethylsiloxanes containing vinyl groups
VOCs	volatile organic compounds
